# Damaging mutations in liver X receptor-α are hepatotoxic and implicate cholesterol sensing in liver health

**DOI:** 10.1038/s42255-024-01126-4

**Published:** 2024-09-25

**Authors:** Sam M. Lockhart, Milan Muso, Ilona Zvetkova, Brian Y. H. Lam, Alessandra Ferrari, Erik Schoenmakers, Katie Duckett, Jack Leslie, Amy Collins, Beatriz Romartínez-Alonso, John A. Tadross, Raina Jia, Eugene J. Gardner, Katherine Kentistou, Yajie Zhao, Felix Day, Alexander Mörseburg, Kara Rainbow, Debra Rimmington, Matteo Mastantuoni, James Harrison, Meritxell Nus, Khalid Guma’a, Sam Sherratt-Mayhew, Xiao Jiang, Katherine R. Smith, Dirk S. Paul, Benjamin Jenkins, Albert Koulman, Maik Pietzner, Claudia Langenberg, Nicholas Wareham, Giles S. Yeo, Krishna Chatterjee, John Schwabe, Fiona Oakley, Derek A. Mann, Peter Tontonoz, Anthony P. Coll, Ken Ong, John R. B. Perry, Stephen O’Rahilly

**Affiliations:** 1 Medical Research Council (MRC) Metabolic Diseases Unit, Institute of Metabolic Science, University of Cambridge, Cambridge, UK; 2 Department of Pathology and Laboratory Medicine, University of California, Los Angeles, CA USA; 3 Newcastle Fibrosis Research Group, Bioscience Institute, Faculty of Medical Sciences, Newcastle University, Newcastle upon Tyne, UK; 4 Institute of Structural and Chemical Biology, Department of Molecular and Cell Biology, University of Leicester, Leicester, UK; 5 Department of Histopathology and Cambridge Genomics Laboratory, Cambridge University Hospitals NHS Foundation Trust, Cambridge, UK; 6 Medical Research Council (MRC) Epidemiology Unit, Institute of Metabolic Science, University of Cambridge, Cambridge, UK; 7 VPD Heart and Lung Research Institute, Dept. Medicine, University of Cambridge, Cambridge, UK; 8 Centre for Genomics Research, Discovery Sciences, BioPharmaceuticals R&D, AstraZeneca, Cambridge, UK; 9 NIHR BRC Core Metabolomics and Lipidomics Laboratory, Metabolic Research Laboratories, Institute of Metabolic Science, University of Cambridge, Cambridge, UK; 10 Computational Medicine, Berlin Institute of Health at Charité - Universitätsmedizin Berlin, Berlin, Germany; 11 Precision Healthcare University Research Institute, Queen Mary University of London, London, UK; 12 NIHR Cambridge Biomedical Research Centre, Cambridge, UK

**Keywords:** Genetics research, Metabolic syndrome, Liver fibrosis, Metabolic syndrome

## Abstract

Liver X receptor-α (LXRα) regulates cellular cholesterol abundance and potently activates hepatic lipogenesis. Here we show that at least 1 in 450 people in the UK Biobank carry functionally impaired mutations in LXRα, which is associated with biochemical evidence of hepatic dysfunction. On a western diet, male and female mice homozygous for a dominant negative mutation in LXRα have elevated liver cholesterol, diffuse cholesterol crystal accumulation and develop severe hepatitis and fibrosis, despite reduced liver triglyceride and no steatosis. This phenotype does not occur on low-cholesterol diets and can be prevented by hepatocyte-specific overexpression of LXRα. LXRα knockout mice exhibit a milder phenotype with regional variation in cholesterol crystal deposition and inflammation inversely correlating with steatosis. In summary, LXRα is necessary for the maintenance of hepatocyte health, likely due to regulation of cellular cholesterol content. The inverse association between steatosis and both inflammation and cholesterol crystallization may represent a protective action of hepatic lipogenesis in the context of excess hepatic cholesterol.

## Abstract (web summary)

LXRα is highly expressed in hepatocytes, where it regulates cholesterol abundance and stimulates lipogenesis. The authors provide evidence in humans and mice that impaired LXRα signalling is hepatotoxic, despite its potent lipogenic actions.

## Main

Liver X receptors (LXRs) are oxysterol-regulated nuclear receptors critical for cellular and organismal cholesterol homoeostasis^1–3^. In hepatocytes, LXRα is the dominant isoform and its activation results in a transcriptional programme that normalizes cellular cholesterol via modulation of cholesterol biosynthesis, uptake and excretion^2,4–7^. LXR agonism also activates reverse cholesterol transport through induction of *ABCG1* and *ABCA1* in the periphery resulting in cholesterol efflux to high-density lipoprotein (HDL) for transport to the liver^8–10^. An additional consequence of LXRα activation is upregulation of hepatic lipogenesis via induction of the lipogenic factors *SREBP1c*, *FASN* and *SCD1*, among others^2^. Thus, in mice, synthetic LXR agonists result in an increase in liver and serum triglycerides that seem largely dependent on hepatocyte LXRα^8,11^.

Initial efforts to translate LXR biology to clinical application focused on using LXR agonism to enhance reverse cholesterol transport to prevent atherosclerotic cardiovascular disease^2,12,13^. However, these efforts have been frustrated by challenges in dissociating the effects of agonism on reverse cholesterol transport from the undesirable effects on hepatic lipogenesis and by species-related effects on lipoprotein metabolism^12,14^. Recently, interest has grown in adopting the converse approach: inhibiting hepatic LXR to suppress lipogenesis and hepatocyte triglyceride accumulation in the context of metabolic dysfunction-associated steatotic liver disease (MASLD), a condition characterized by hepatic steatosis and cardiometabolic risk factors without significant alcohol consumption^15^. In a proportion of individuals, neutral lipid can be stored in the liver without significant hepatic inflammation. In a minority, steatosis is associated with the development of inflammation, so-called metabolic dysfunction-associated steatohepatitis (MASH), which can result in fibrosis, cirrhosis and end-stage liver disease^16,17^. While the mechanistic basis of this heterogeneity is not clear, excess hepatic triglyceride likely plays a causal role in MASH development and progression^18–20^.

It is therefore reasonable to ask whether suppression of lipogenesis via inverse agonism of LXR might be beneficial in MASLD. Treatment with LXR-inverse agonists reduce steatosis, inflammation and fibrosis in rodent models^15,21,22^ and early-stage clinical trials of LXR-inverse agonists for hypertriglyceridaemia and MASLD are in progress^23^. However, increased hepatic cholesterol has been observed in MASLD and is a putative risk factor for MASH^24–26^. Moreover, several mouse models with hepatic cholesterol accumulation due to genetic perturbation of key regulators of cholesterol homoeostasis (including LXRα) exhibit evidence of liver injury^27–30^. Increasing dietary cholesterol content increases the hepatic inflammation and fibrosis associated with high-fat diets in mice^31,32^ and has been shown to stimulate a fibrogenic pathway in liver via TAZ-dependent activation of the Indian hedgehog pathway^33^. Thus, impaired hepatic cholesterol clearance may undermine the therapeutic utility of LXR-inverse agonists in MASLD.

It is increasingly recognized that human genetics can assist drug discovery by the validation of drug targets and the prediction of unwanted effects of target engagement^34,35^. Given the translational interest in LXR in MASLD and other facets of cardiometabolic health we sought to determine the effects of damaging mutations in LXRα, the dominant LXR isoform in the liver, on human cardiometabolic health. We find that carriers of proven damaging mutations in LXRα are associated with evidence of hepatotoxicity despite evidence of reduced lipogenesis. We demonstrate that knock-in mice carrying a damaging, dominant negative mutation in LXRα develop liver inflammation and severe fibrotic liver injury despite marked reduction in liver triglycerides and steatosis. Together our results highlight the essentiality of intact LXRα signalling and hepatic cholesterol homoeostasis for liver health.

## Results

### Identification of damaging mutations in LXRα in UKBB

**Fig. 1:**
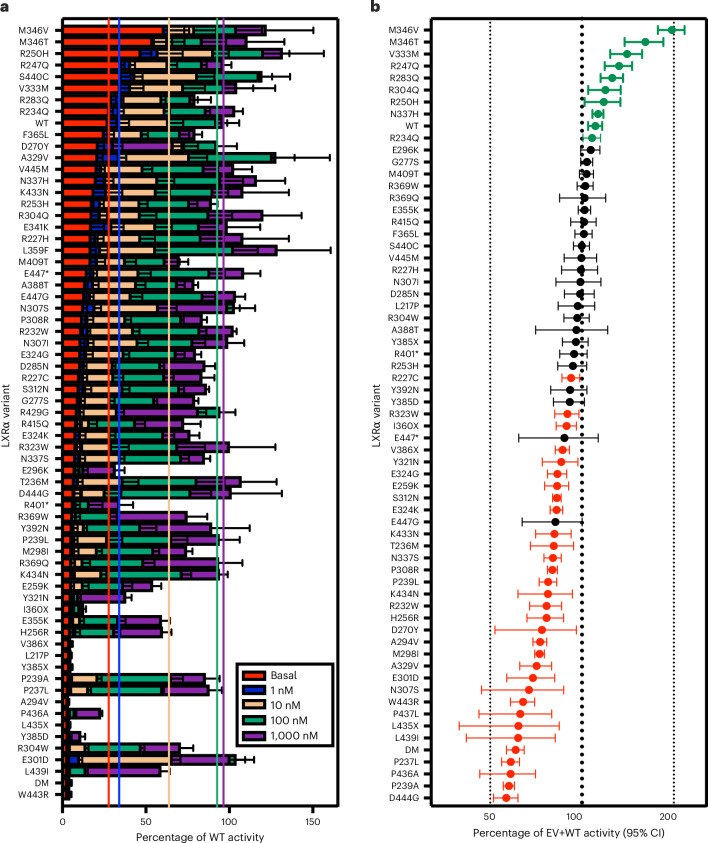
Identification of loss of function and dominant negative mutations in the ligand-binding domain of LXRα carried by participants in population biobanks. We characterized 65 coding variants in the ligand-binding domain of LXRα in two separate assays. **a**, Stacked bar chart illustrating LXRα mutant activity. HEK293 cells were transfected with an LXR luciferase reporter construct and WT or mutant LXRα. At 4 h after transfection, cells were treated with indicated concentration of the LXRα agonist T0901317 and luminescence was measured as an index of LXRα activity 20 h later. The dashed coloured lines indicate average activity of WT LXRα at the corresponding concentration of T0901317. Statistical analysis of mutant versus WT activity was assessed by two-way ANOVA with Geisser–Greenhouse correction and multiple comparison controlling for false discovery rate (FDR) at each ligand concentration by two-stage step-up method of Benjamini, Krieger and Yekutieli. FDR-corrected *P* values are presented in Supplementary Table 1. Each mutant was tested in a minimum of three independent experiments, resulting in a total of 25 independent experiments conducted across seven individual batches. The height of bars and error bars represent mean ± s.d. **b**, Forest plot of mutant LXRα activity in a co-expression assay. WT and mutant LXRα and an LXR luciferase reporter construct were transfected into HEK293 cells. At 24 h later luminescence was measured as an index of LXRα activity. Assuming normal distribution, significant increases and decreases according to a two-tailed one-sample *t*-test are depicted in green and red, respectively. *n* = 4–8 independent experiments per variant were performed on different days in the same cell line. Dots and error bars represent mean ± 95% CIs. EV, empty vector; DM, an artificial double mutant that is strongly DN. Source data

**Extended Data Fig. 1:**
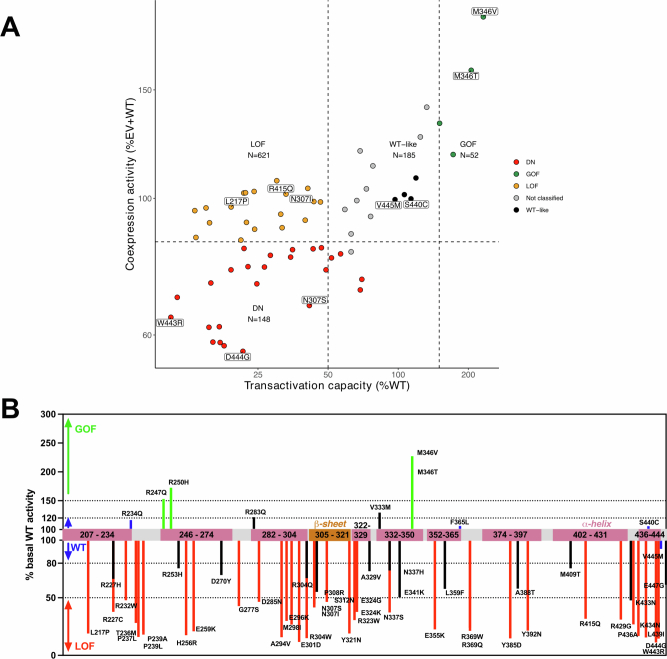
Summary of LXRα mutant effects on receptor function. **A:** Scatter plot demonstrating the relationship between transactivation capacity and co-expression activity for characterized mutations. Each dot is an individual variant, green variants are gain of function mutations, black are wild-type like, orange are loss of function without evidence of significant dominant negativity and red dots are dominant negative mutations. Grey dots are mutations which are intermediate and we not assigned to a class for the purpose of burden testing. The ‘N’ numbers represent the number of carriers in UK biobank. **B:** Schematic illustrating the effect of characterized LXR α mutants according to their position in the ligand binding domain of LXRα. Red, green and blue bars denote loss of function, gain of function and wild-type like mutations, respectively.

To identify human carriers of damaging mutations in LXRα, we interrogated exome sequencing data from 454,756 UK Biobank (UKBB) participants. We focused on the ligand-binding domain as experience with naturally occurring mutations in other nuclear receptors (for example PPARγ and TRα/β) indicates a strong enrichment of pathological variants in this region^36–38^. We selected (Methods) a total of 63 rare variants (minor allele fraction (MAF) < 0.1%, 57 predicted deleterious missense variants and six protein-truncating variants (PTVs) near the C terminus, all present in heterozygosity) in the ligand-binding domain of LXRα (Supplementary Table 1) for characterization in two different assays of nuclear receptor function: (1) a transactivation assay; and (2) a co-expression assay examining the ability of candidate LXRα variants to impair co-transfected wild-type LXRα as an index of dominant negative activity. We identified 23 mutations with evidence of dominant negativity (DN; *n*(carriers) = 162, cumulative MAF = 0.04%), 20 mutations with evidence of impaired transactivation without significant dominant negative activity (loss of function (LOF); *n*(carriers) = 642, cumulative MAF = 0.14%) and four gain of function (GOF) mutations (*n*(carriers) = 52, cumulative MAF = 0.01%) (Fig. 1 and Supplementary Tables 1 and 2). DN mutations were less prevalent in UKBB than LOF, and most dominant negative mutations exhibited only modest dominant negative activity (Fig. 1 and Extended Data Fig. 1). We characterized an additional two variants in the Fenland study (Methods) that were not present in UKBB, both of which were categorized as DN. Our results demonstrate that damaging mutations in LXRα are prevalent in the general population.

### Co-factor association and heterogeneity in variant effect

**Fig. 2:**
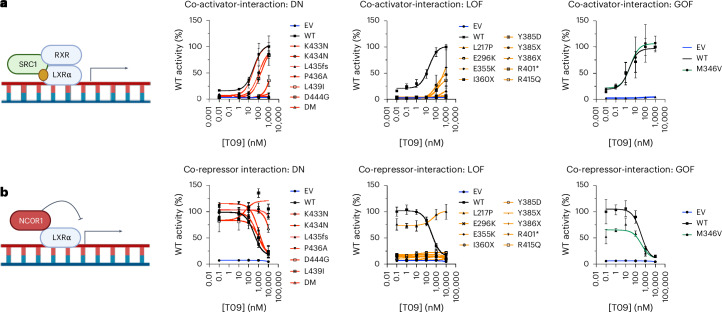
Co-repressor association is a key determinant of heterogeneity of mutant impact. **a**,**b**, We used mammalian two-hybrid assays to assess the effect of mutations in the ligand-binding domain of LXRα on co-activator (**a**) and co-repressor association (**b**). HEK293 cells were co-transfected with plasmids expressing LXRa-VP16, either SRC1-GAL4 (**a**) or NCOR1-GAL4 (**b**) fusion constructs with a UAS-TK luciferase reporter construct. At 4 h after transfection, cells were treated with the indicated concentration of T0901317 (T09) for 20 h before luminescence was assayed as an index of VP16 and GAL4-conjugate association. Mutants were analysed in three batches each consisting of three independent experiments and are presented as normalized WT luciferase activity (%) in each batch. The mutations are separated by their classification for clarity of presentation and, where possible, are presented alongside the corresponding WT control according to experimental batch. The DN and LOF groups had mutants present in two and three batches. Therefore, the WT control values presented in these panels are the averages of six independent experiments for the dominant negative group and nine independent experiments for the LOF group. The curves represent lines of best fit generated in a three-parameter model conducted in Prism (GraphPad). For the purposes of curve fitting and plotting on a log axis, the untreated condition is plotted as an order of magnitude less than the lowest dose of agonist used. Formal hypothesis testing of mutation class on co-repressor/co-activator association versus WT was conducted using a mixed-effects model with a post hoc Dunnet’s test implemented in R (Methods). *P* values from these analyses are presented in Supplementary Table 3. Symbols and error bars represent mean ± s.e.m. Source data

Nuclear receptors can repress or activate gene expression, dependent on the repertoire of bound co-activators and co-repressors. Disruption in co-repressor and co-activator association provides an obvious mechanistic basis for the effects of impactful variants on LXRα function. We used mammalian two-hybrid assays to determine whether differences in co-repressor or co-activator association were differentially affected by LOF and DN mutations. Both LOF and DN mutations exhibited evidence of impaired co-activator (SRC1) association in response to increasing doses of synthetic LXRα agonist (Fig. 2a). In contrast, co-repressor association (NCOR1) seemed relatively preserved across DN mutations but markedly impaired in all but one (p.L217P) of the LOF variants studied (Fig. 2b). To formally compare LOF and DN mutations to wild-type (WT) we fitted mixed-effects models with mutation class and agonist dose as interacting covariates (Methods). Co-activator association was significantly impaired in both DN and LOF mutation groups across the majority of agonist doses tested, whereas only LOF mutations were significantly different from WT when co-repressor association was analysed (Supplementary Table 3). In the single GOF mutation tested, M346V, co-activator association seemed comparable with WT LXRα, whereas co-repressor association was reduced. Together, these findings suggest that alteration in co-activator and co-repressor affinity of LXRα may be key determinants of variant effect.

### GOF variants alter protein stability and co-factor affinity

To further investigate the properties of the GOF receptors we explored the ability of the receptor ligand bingding domain (LBD) to interact with co-repressor and co-activator peptides in vitro. The strongest GOF mutation p.M346V exhibited twofold reduced affinity for co-repressor peptide using a fluorescence anisotropy assay (Supplementary Fig. 1a). Unexpectedly, the p.M346V mutant also showed a reduced affinity for co-activators in the presence of agonist. This contrasts with the cell-based two-hybrid assays, which showed preserved co-activator association with the mutant receptor (Supplementary Fig. 1b), though it is notable that in cells the GOF was most significant in the absence of added ligand (Fig. 1a) and thus seems likely to be caused by perturbation in co-repressor interaction.

The p.M346V mutation is distant from the co-repressor/co-activator recruitment surface. Therefore, we performed thermal denaturation studies, monitored by circular dichroism, to determine melting temperatures and assess whether the mutation might affect LBD stability. Both mutants p.M346V and p.M346T showed an increase in the melting temperature compared with WT and these melting temperatures do not increase in the presence of the agonist (Supplementary Fig. 1c). This indicates that the core of the unliganded mutant proteins is more stable than the WT. It is not clear how this leads to reduced co-repressor binding, but it is possible that it results in helix 12 (H12) of the receptor adopting a position that disfavours co-repressor interaction.

M346 is far from the co-repressor and co-activator binding site and there are no significant structural changes predicted by AlphaFold (Supplementary Fig. 2)^39^. This suggests that the mutants may cause allosteric changes in the conformational equilibrium of the ligand-binding domain (Supplementary Fig. 2a–c), in agreement with the observed differences in stability. Methionine 346 is surrounded by hydrophobic residues and near two aromatic amino acids (phenylalanine 342 and phenylalanine 356) that would interact with the sulfur group and the methyl of the methionine, respectively (Supplementary Fig. 2a–c). Valine is a smaller hydrophobic residue that might preserve some of these interactions. On the other hand, threonine has a polar side chain that could potentially interact with serine 303, which in turn could also interact with serine 343. This hydrogen bond network might explain the increase in stability of the p.M346T mutant (Supplementary Fig. 2c). Together, these findings suggest that alteration in stability of the mutant LXRα can affect co-activator and co-repressor affinity and may be a determinant of variant effect.

### Cardiometabolic consequences of damaging mutations in LXRα

**Extended Data Fig. 2:**
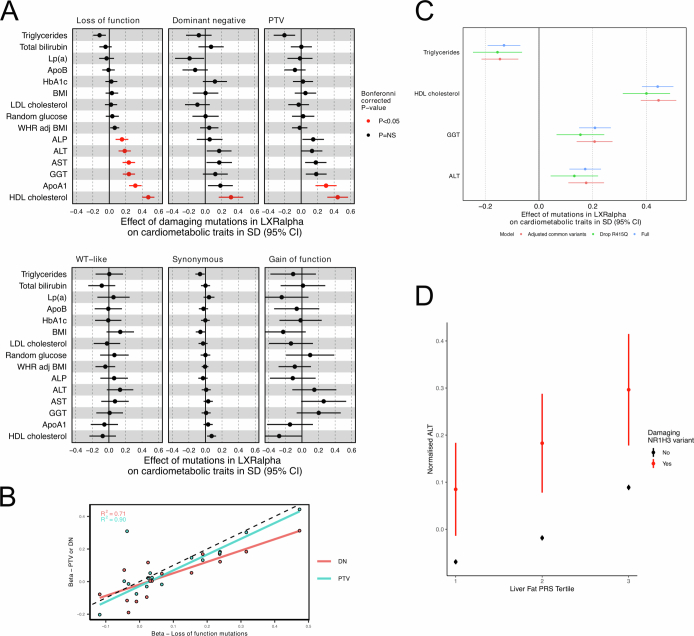
Effects of coding variants in LXRα on cardiometabolic traits. **A:** The effect of coding variants in LXRα on a panel of pre-selected cardiometabolic traits. Each dot represents the effect of a damaging mutation on the trait in standard deviation, the error bars represent 95% confidence intervals, each derived from a generalized linear model (see methods). Red points indicate statistical significance after adjustment for multiple testing (Bonferroni-corrected P-value – 3.9×10^−4^), NS = non-significant. **B:** Scatter plot demonstrating the relationship between the average effect of loss of function mutations and all dominant negative (DN) or protein truncating variants (PTV) on cardiometabolic trait. Each dot plots the effect of the Loss of function mutations on a single trait against either PTVs (blue) or DN (red). The dashed line represents y = x where there would be perfect agreement between the effects of Loss of function mutations and DN/PTV. **C:** Forest plot of sensitivity analyses. ‘Full’ illustrates the effect of damaging mutations in LXRα (LOF or DN or PTV) from the primary analysis, Drop R415Q – analysis after removing the most common rare variant (R415Q, N(carriers in all UKBB) = 565) or ‘Adjusted common variants’ - adjusting for common variants in the region significantly associated with the listed traits of interest. **D:** Scatter plot of scaled, centred log normalized ALT values (0 represents mean ALT, units are standard deviation) according to tertile of liver fat polygenic risk score (PRS) and presence/absence of a damaging variant in LXRα. Error bars represent 95% confidence intervals. LP(a) – Lipoprotein-a, LDL – low density lipoprotein, BMI – body mass index, WHR adj BMI – waist hip ratio adjusted for BMI, ALP – alkaline phosphatase, ALT – alanine aminotransferase, GGT – gamma glutamyl transferase, AST – aspartate aminotransferase, ApoA1 – Apolipoprotein A1, HDL – high density lipoprotein. Source data

To determine the effect of LXRα variants on human health, we compared carriers of experimentally characterized variants, synonymous and bioinformatically predicted PTVs to non-carriers in UKBB using gene burden testing (Extended Data Fig. 2a and Supplementary Table 6). Carriers of LOF mutations that did not exhibit significant dominant negative activity had higher serum HDL cholesterol, apolipoprotein A1 and liver enzymes (Extended Data Fig. 2a and Supplementary Table 6). In general, directionally consistent findings were observed for both DN and PTVs (Extended Data Fig. 2a,b). There was no evidence of a greater effect of DN mutations across the traits tested, though this may reflect lack of statistical power and that most variants exhibited only modest dominant negative activity (Extended Data Fig. 1a). When aggregated, rare (MAF < 0.1%) synonymous variants, which were included as a negative control mask, did not exhibit any significant associations after adjustment for multiple testing (Extended Data Fig. 2a and Supplementary Table 6).

**Fig. 3:**
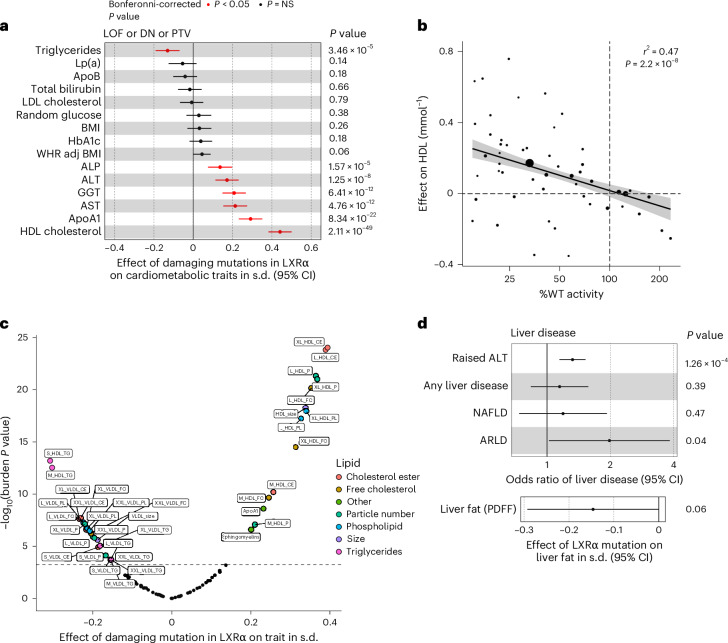
Damaging mutations in LXRα are associated with elevated liver enzymes despite beneficial effects on lipid metabolism. **a**, Forest plot illustrating the effects of carriage of a damaging mutation in LXRα (DN or LOF bioinformatically predicted PTVs) on a selection of cardiometabolic traits in European participants in the UKBB. Each dot represents the effect of a damaging mutation on the trait in s.d., the error bars represent 95% CIs, each derived from a generalized linear model (Methods). Red points indicate statistical significance after correction for multiple testing using the Bonferroni method (*P* < 3.9 × 10−^4^), NS, not significant. **b**, Scatter-plot illustrating the relationship between LXRα mutant activity and HDL cholesterol. Each dot represents an individual mutation, the size of the dot is proportional to number of carriers included in the analysis and weight in the model. The solid black line and grey band represent the estimated mean (± 95% CIs) effect of changes in LXRα activity on HDL cholesterol derived from a weighted regression analysis. **c**, Volcano plot illustrating the effect of damaging mutations (LOF or DN or PTV) in LXRα on lipoprotein particle size and composition assessed by nuclear magnetic resonance using the Nightingale health platform in UKBB. A full list of abbreviations for lipid measurements in **c** are available in Supplementary Table 10. The dotted line represents the Bonferroni-adjusted *P* value cutoff (*P* < 5.7 × 10^−^^4^). **d**, Forest plot illustrating the effect of damaging mutations in LXRα on liver disease and liver fat in UKBB. Each dot represents the effect of a damaging mutation on the trait in s.d. if continuous or log(odds) if binary, the error bars represent 95% CIs. LP(a), lipoprotein-a; BMI, body mass index; WHR adj BMI, waist hip ratio adjusted for BMI; ALP, alkaline phosphatase; GGT, γ glutamyl transferase; ApoA1, apolipoprotein A1; NAFLD, non-alcoholic fatty liver disease; ARLD, alcohol-related liver disease. *P* values are two-sided. Burden *P* values were calculated in STAAR (Methods). Source data

Given the comparability of effects across all classes of damaging mutations, we collapsed LOF, DN and PTVs to a single damaging mask to maximize statistical power (*n*(variants) = 70, *n*(carriers) = 1,029, cumulative MAF = 0.23%). A notable elevation of serum HDL cholesterol was observed in carriers of damaging LXRα variants (Fig. 3a and Supplementary Table 6) and we observed a dose–response relationship between LXRα variant activity and variant effect on HDL cholesterol (Fig. 3b and Supplementary Table 7). Damaging mutations in LXRα were associated with significant reductions in serum triglycerides, consistent with loss of the known lipogenic effects of LXRα, but an elevation in serum liver enzymes (Fig. 3a and Supplementary Table 6). These associations were robust to exclusion of the most common damaging variant, R415Q, and to adjustment for regional common variant signals (Extended Data Fig. 2c and Supplementary Tables 8 and 9).

In view of the effects of damaging LXRα variants on serum lipids, we characterized their impact on lipoprotein composition using NMR metabolomic data available in a subset (*n* = ~280,000) of exome-sequenced participants in the UKBB. We observed elevations in cholesterol ester content in circulating HDL particles, increased HDL particle size and reduced HDL triglycerides (Fig. 3c and Supplementary Tables 10 and 11). This pattern is suggestive of relative CETP deficiency, an enzyme catalysing transfer of cholesterol esters from HDL to triglyceride rich lipoproteins in exchange for triglyceride and a known LXRα target gene^40^. Very-low-density lipoprotein (VLDL) triglyceride content was also reduced (Fig. 3c), consistent with the effects of LXRα on hepatic lipogenesis that had been described in preclinical models^2^.

Damaging mutations in LXRα were associated with higher liver enzymes, suggestive of subclinical hepatotoxicity. We explored this relationship further and found carriers in UKBB had a 32% increased risk of clinically significant elevations in alanine aminotransferase (ALT) and a nominal increase in risk of clinically diagnosed alcohol-related liver disease though this observation is based on only nine affected carriers and should be treated with caution (Fig. 3d and Supplementary Table 12). Liver fat measurements were only available in a small subset of UKBB, nevertheless we observed a trend to reduction in liver fat despite the elevation in serum liver enzymes (Fig. 3d and Supplementary Table 12). Rare variant associations with liver function tests and alcohol-related liver disease were consistent when using an independently established computational pipeline and an additional PTV-augmented model in 462,096 UKBB whole genomes of European genetic ancestry^41^ (Supplementary Tables 13–16).

We reasoned that the effects of damaging LXRα variants could depend on underlying disposition to liver fat accumulation, potentially exerting protective effects in those otherwise predisposed to increased liver fat. To test this, we derived a polygenic risk score using genome-wide significant variants single-nucleotide polymorphisms from a meta-analysis of >60,000 liver fat measurements and historical NAFLD diagnoses across three cohorts. As expected, this risk score was strongly associated with liver fat in UKBB (normalized proton density fat fraction (PDFF): *β* = 0.22 (95% CI 0.21–0.23), *P* < 4.94 × 10^−324^) and increased ALT (0.077 (95% CI 0.074–0.081), *P* < 4.94 × 10^−324^). Notably, there was no evidence of an interaction between genetic risk score and LXRα carrier status with respect to their association with ALT (*P* = 0.35) (Extended Data Fig. 2d).

### DN LXRα mutations are hepatotoxic in mice

**Extended Data Fig. 3:**
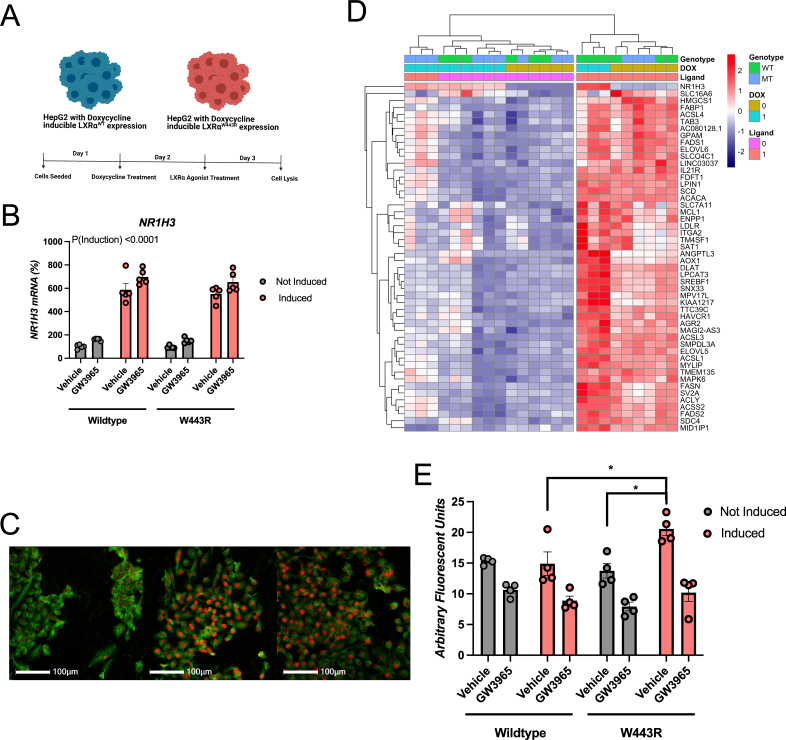
A naturally occurring variant in LXRα exerts dominant negative effects in cultured hepatocytes. **A:** To demonstrate dominant negative effects of LXRα^W443R^ in a relevant cell type we generated HepG2 cell lines stably transfected with a Doxycycline-inducible transgene expressing either a wild-type LXRα isoform (WT) or a mutant LXRα^W443R^ (MT or W443R). The experimental paradigm is summarized in (A), 24 hours after seeding cells were treated with doxycycline to induce LXRα expression, 24 hours later LXR agonist GW3965 (1000 nM) was added and cells were lysed 24 hours later. **B:** Expression of *NR1H3* mRNA (encoding LXRα) in the paradigm described in (A). N = 5 independent experiments. Two-sided P values are displayed from 3-way ANOVA with post hoc Holm-Šídak test. Error bars represent mean ± SEM **C:** Representative immunofluorescent images of non-transduced HepG2 cells and WT or MT/W443R HepG2 cells treated with doxycycline stained for Beta-Tubulin, LXRα and DAPI. Enhanced LXRα nuclear immunoreactivity can be seen in the transduced cells consistent with induction of LXRα. This experiment was performed twice with consistent results **D:** Heatmap illustrating expression of GW3965 regulated genes in an RNA-seq experiment after treatment of cells in the paradigm outlined in (A). Ligand treated cells with overexpression of LXRα^W443R^ cluster with non-ligand treated conditions, indicative of the inhibitory effects of LXRα^W443R^ on LXR-dependent signalling. Furthermore, induction of canonical LXR-regulated genes, including *FADS1, SCD, ANGPTL3, LPCAT3, FASN, MYLIP and SREBF1*, is impaired by induction of LXRα^W443R^, consistent with dominant negative actions of LXRα^W443R^. N = 3 independent experiments. **E:** We further validated the dominant negative actions of LXRα^W443R^ in hepatocytes in an LDL uptake paradigm. Following the experimental paradigm illustrated in (A), cells were treated with Dil-labelled LDL for 4 hours before cells were lysed and fluorescence measured as an index of LDL uptake. LXRα^W443R^ induction was capable of increasing LDL uptake in the basal state, counteracting the previously described effects of LXRα in this setting. Error bars represent mean ± SEM. Analysis was conducted by three-way ANOVA with post hoc testing undertaken using the Holm-Šídak method. *P = 0.01 Induced vehicle treated wildype versus Induced vehicle treated W443R, P = 0.02 Not-induced vehicle treated W443R versus Induced W443R Vehicle treated, N = 4 independent experiments. Figure 3a was created with Biorender. Source data

Intrigued by the paradoxical effects of damaging mutations in human LXRα on indices of hepatic lipogenesis and liver enzymes in humans, we explored the mechanistic basis of these effects using a mouse model. We used CRISPR-Cas9 to generate a knock-in mouse model of one of the most potent DN mutations that we studied, LXRα W443R (W441R in mice), which was found in a single carrier in the Fenland study. This variant substitutes an aromatic residue in helix 12 of the ligand-binding domain that is essential for ligand-dependent activation of the receptor^42^. The AlphaFold model of p.W443R LXRα–LBD clearly shows how the change of tryptophan 443, a bulky hydrophobic residue that interacts directly with the agonist, for an arginine, a positively charged residue, changes the conformation of helix 12 displacing it from the active position (Supplementary Fig 3b, magenta arrows). The mutant repressed LXR-regulated genes and impaired low-density lipoprotein (LDL) uptake when overexpressed in a hepatoma cell line (Extended Data Fig. 3).

**Extended Data Fig. 4:**
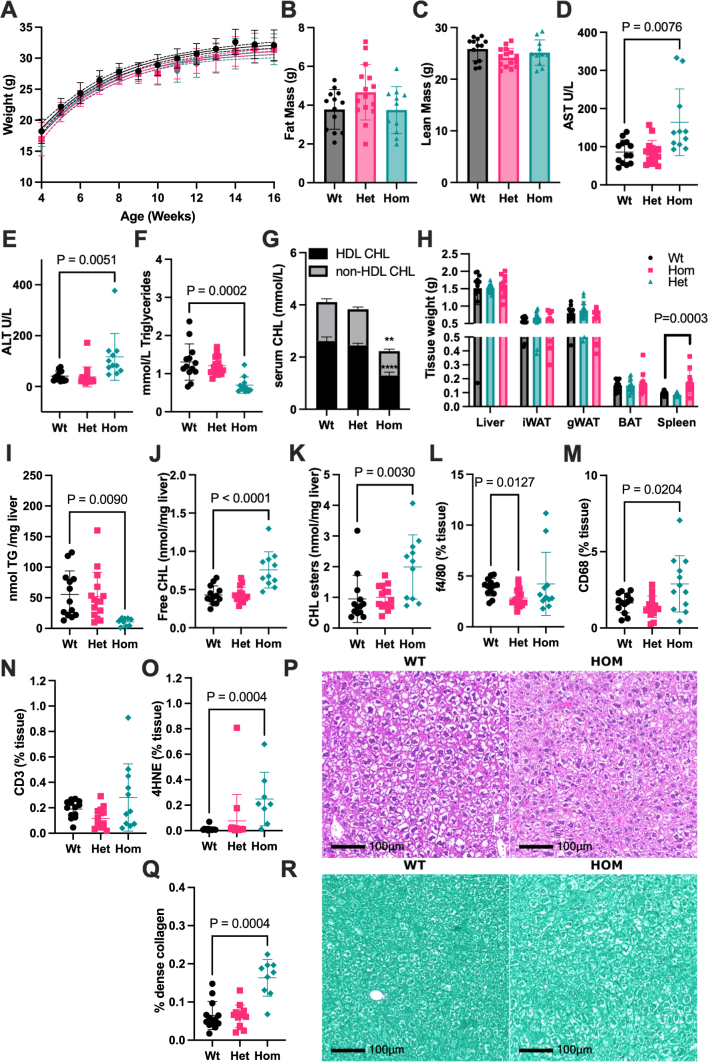
Hepatotoxicity of a dominant negative LXRα mutation in mice fed a low fat diet. 8-week-old LXRα^+/+^ (Wt, N = 13), LXRα^+/W441R^ (Het, N = 15) and LXRα^W441R/W441R^ (Hom, N = 11) mice were placed on a low fat (12% by Kcal), high-sucrose (34.1% by weight) diet for 8 weeks when mice were euthanised and blood and tissues were collected for downstream analyses. **A:** Body weight curves (mean and 95% confidence intervals). **B-C:** Lean and fat mass determined by Echo MRI at 16 weeks of age. **D-G:** Serum biochemistry from terminal bleeds, in **(G)** Total cholesterol is separated into HDL and non-HDL fractions, ****P < 0.0001, HDL cholesterol, **P = 0.001 non-HDL cholesterol. **H:**Tissue weights, **I-K:** lipid measurements in liver lysate normalized to liver weight. **L-O:** Immunohistochemical staining for indicated markers was performed and quantified using HALO image analysis platform (Indica labs). Representative images of H&E staining are shown in (**P**). Livers were stained for collagen using picrosirius red (PSR) stain and quantified by HALO (**Q**). Representative images of PSR staining are shown in (**R**). **A:** Body weight curves were compared by mixed-effects model was used with post hoc Holm-Šídak test. **B-O, Q:** Analysis was performed using Kruskal–Wallis test with Dunn’s multiple comparison test or ordinary one-way ANOVA with Holm-Šídak multiple comparison, based on the distribution of the data. Comparisons were made between Wt and Het and Wt and Hom, only P < 0.05 are detailed. All reported P values are two-sided. All data are presented as mean ± SD, except Panel G, where mean ± SEM is represented. Source data

LXRα^W441R/W441R^ mice fed a low-fat, low-cholesterol diet for 8 weeks were comparable with their WT littermate controls in body weight and composition at 16 weeks of age (Extended Data Fig. 4a–c). Consistent with some features of human carriers of damaging LXRα mutations they had modest elevations in circulating ALT and aspartate aminotransferase (AST) (Extended Data Fig. 4d,e), despite reductions in serum and liver triglycerides (Extended Data Figs. 4f and 4i). In keeping with the role of LXRα in hepatic cholesterol sensing and disposal, free cholesterol and cholesterol esters were elevated in the liver of homozygous knock-in mice (Extended Data Figs. 4j and 4k). Serum HDL cholesterol was reduced (Extended Data Fig. 4g), in line with known species differences in HDL cholesterol metabolism between mice and humans. LXRα^W441R/W441R^ mice also exhibited evidence of increased lipid peroxidation and fibrosis, as assessed by 4-HNE and picrosirius red (PSR) staining, respectively. Inconsistent effects on immunohistochemical markers of hepatic macrophages were observed (Extended Data Fig. 4l–r). There was no gross evidence of any extra-hepatic phenotype at necropsy, except for splenomegaly (Extended Data Fig. 4h).

**Fig. 4:**
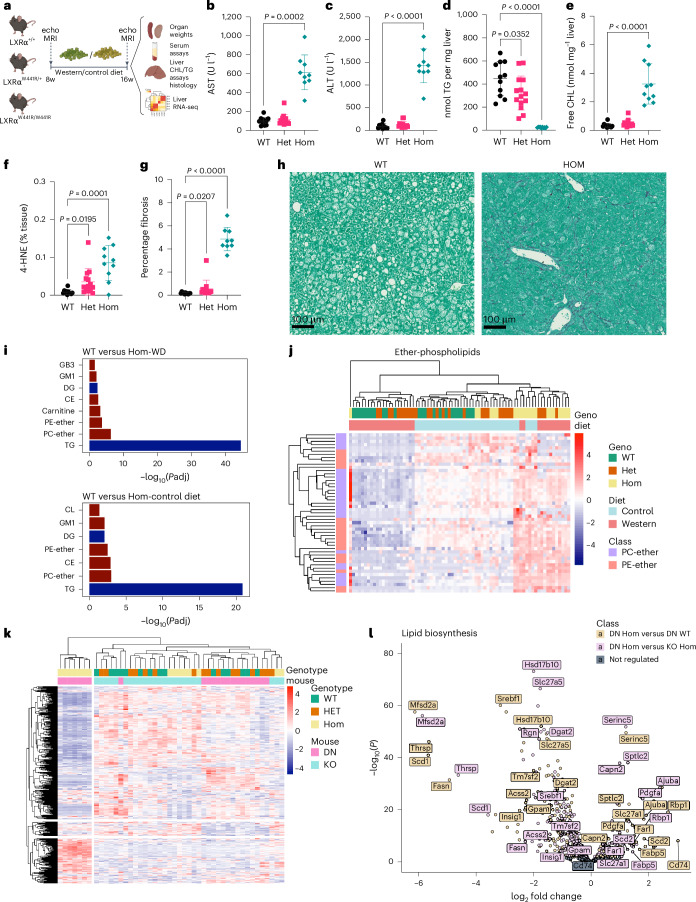
Damaging mutations in LXRα cause liver injury in mice exposed to western diet despite suppression of lipogenesis. **a**, The 8-week-old WT (LXRα^+/+^ or DN:WT, *n* = 15), heterozygous (LXRα^+/W441R^ or DN:HET, *n* = 19) and homozygous (LXRα^W441R/W441R^ or DN:HOM, *n* = 14) male mice were fed a western diet (WD) for 8 weeks. Created with biorender.com. **b**,**c**, After 8 weeks of western diet, 10 WT, 14 heterozygous and 10 homozygous mice were assessed for serum AST (**b**) and ALT (**c**) levels. **d**,**e**, Livers from these same mice were homogenized and assessed for level of triglycerides (**d**) and free cholesterol (**e**). **f**,**g**, Livers were also stained for 4-HNE (**f**) and collagen using PSR stain (**g**) and quantified by HALO. **h**, Representative images of PSR staining. We used mass spectrometry to assess the lipidome of WD and control diet-fed mice. **i**,**j**, Enrichment of lipid classes (**i**) and a heatmap focused on ether phospholipids (**j**). In a separate study, we repeated the experiment as in **a**, with WT (LXRα^+/+^ or KO:WT, *n* = 8), heterozygous (LXRα^+/−^ or KO:HET, *n* = 8) and homozygous (LXRα^−/−^ or KO:HOM, *n* = 8) mice. After killing at 16 weeks, RNA was extracted from the livers of all three genotypes from both studies and sequenced. **k**,**l**, Heatmap of RNA-seq expression data (*n* = 8) showing sample clustering based on the genes of Lipid Biosynthetic Process GO-annotation pathway (GO:0008610) (**k**), volcano plot of same genes (**l**). Two-sided *P* values are reported from Kruskal–Wallis test with Dunn’s multiple comparison test or ordinary one-way ANOVA with Holm–Šídak multiple comparison, based on the distribution of the data (**b**–**g**). All data are presented as mean ± s.d. CHL, cholesterol; TG, triglyceride; MG, monoglyceride; SM, sphingomyelin; S, sulfatides; GB3, GB gangliosides; CL, cardiolipin; GM1, GM1 gangliosides; DG, diglycerol; CE, cholesterol ester; PE, phosphatidylethanolamine; PC, phosphatidylcholine. Source data

**Extended Data Fig. 5:**
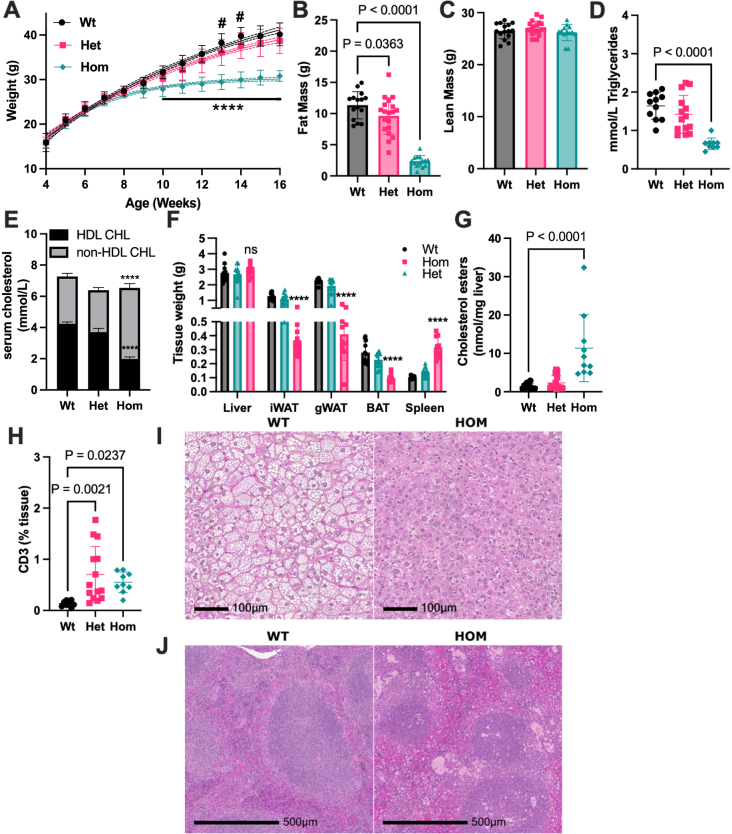
Effects of a dominant negative LXRα mutation in mice fed a western diet for 8 weeks. 8-week-old LXRα^+/+^ (Wt, N = 15), LXRα^+/W441R^ (Het, N = 19) and LXRα^W441R/W441R^ (Hom, N = 14) mice were placed on a high-fat (42.7% by Kcal), high-sucrose (34.1% by weight), high cholesterol (0.2%) diet (western diet) for 8 weeks then euthanised and blood and tissues were collected for downstream analyses. **A:** Body weight curves (mean and 95% confidence intervals). ****P < 0.0001, Wt vs Hom, # P = 0.02 Wt vs Het week 13, P = 0.01 Wt vs Het week 14. **B-C:** Fat and lean mass determined by Echo MRI at 16 weeks of age. For 11 wild-type, 14 heterozygous and 10 homozygous mice we performed downstream analysis including: serum biochemistry from terminal bleeds (**D-E**). In **(E)** total cholesterol is disaggregated into HDL and Non-HDL cholesterol. The asterixis represent significant differences in HDL cholesterol and non-HDL cholesterol, ****P < 0.0001 versus Wild-type, HDL cholesterol is significantly reduced, non-HDL cholesterol is significantly increased. **F:** Tissue weights, ****P < 0.0001 **G:** Cholesterol Esters measured in liver lysate **H:** Immunohistochemical staining for CD3 was performed and quantified using HALO image analysis platform (Indica labs). Representative images of H&E staining of liver and spleen are shown in **I** and **J**, respectively. In **(A)** a mixed-effects model was used to account for repeated measures with post hoc Holm-Šídak test. In (**B-H)**, analysis was performed using Kruskal–Wallis test with Dunn’s multiple comparison test or ordinary one-way ANOVA with Holm-Šídak multiple comparison, based on the distribution of the data. Comparisons were made between Wt and Het and Wt and Hom, only P < 0.05 are detailed. All P values reported are two-sided. Data are presented as mean ± SD, except panel E, where mean ± SEM is represented. Source data

In contrast with the relatively modest phenotype observed on a low-fat, low-cholesterol diet, after 8 weeks of exposure to a western diet (0.2% cholesterol) LXRα^W441R/W441R^ mice had attenuated weight gain (Extended Data Fig. 5a–c) and exhibited a marked elevation in circulating levels of ALT and AST despite suppression of serum and hepatic triglycerides (Fig. 4a–d and Extended Data Fig. 5d). Compared with WT mice, the homozygous knock-in mice exhibited ~eightfold and ~tenfold increase in esterified and free cholesterol, respectively (Fig. 4e and Extended Data Fig. 5g). Histopathological analysis of livers demonstrated xanthogranulomatous inflammation occurring despite the striking absence of steatosis in LXRα^W441R/W441R^ mice with evidence of an allele-dependent increase in lipid peroxidation and hepatic fibrosis (Fig. 4f–h). Extra-hepatic phenotypic assessment was notable for splenomegaly and reductions in fat pad mass (Extended Data Fig. 5f). Histological analysis of the spleen demonstrated accumulation of lipid-laden macrophages, possibly accumulating following clearance of excess tissue cholesterol (Extended Data Fig. 5J).

Given the key regulatory function of LXRα in liver lipid metabolism, we characterized the hepatic lipidome of our knock-in mice. The most notable observation was a marked downregulation of hepatic triglyceride species in LXRα^W441R/W441R^ mice on a control and western diet consistent with loss of the lipogenic actions of LXRα (Fig. 4i). On both diets, there was an upregulation of ether phospholipids and on the western diet, carnitine species increased, suggesting that available fatty acids may be diverted to peroxisomes and mitochondria for utilization (Fig. 4i,j).

### LXRα^−/−^ mice develop milder liver injury than LXRα^W441R/W441R^ mice

**Extended Data Fig. 6:**
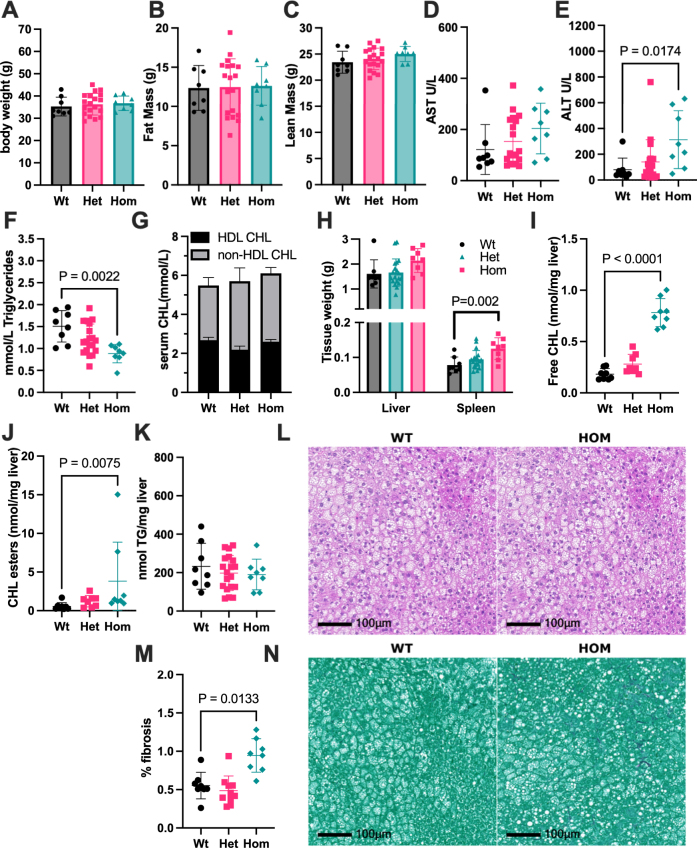
Hepatotoxicity in LXRα knockout mice fed a western diet. 8-week-old wild-type LXRα^+/+^ (Wt, N = 8), heterozygous LXRα^+/-^ (Het, N = 19) and homozygous LXRα^-/-^ (Hom, N = 8) mice were placed on a high-fat (42.7% by Kcal), high-sucrose (34.1% by weight), high cholesterol (0.2%) diet (western diet) for 8 weeks then euthanised and blood and tissues were collected for downstream analyses. Unless specified, all mice were assessed for: **A**: Endpoint weight measurements **B-C:** Fat and lean mass assessed by Echo MRI at 16 weeks of age. **D-G**: Serum biochemistry from terminal bleeds. **H:** Selected organ weights. **I-J:** Free cholesterol (CHL) and CHL ester measurements in liver lysate normalized to organ weight are shown for N = 8 mice of each genotype. **K:** Triglyceride (TG) measurements in liver lysate normalized to organ weight are shown for all mice. Representative images of H&E staining are shown in (**L**). Livers from wild-type (N = 8), heterozygous (N = 12) and homozygous (N = 8) mice were stained for collagen using picrosirius red (PSR) stain and quantified by HALO (**M**). Representative images of PSR staining are shown in (**N**). In (**A-K, M)**, analysis was performed using Kruskal–Wallis test with Dunn’s multiple comparison test or ordinary one-way ANOVA with Holm-Šídak multiple comparison, based on the distribution of the data. All data are presented as mean ± SD, except G where mean ± SEM is represented. Source data

LXRα^−^^/−^ mice have previously been shown to develop fibrotic liver injury after prolonged exposure to a very-high-cholesterol diet (2% cholesterol diet for 9 months)^27^. To test whether the repressive actions of the W441R variant exacerbated liver injury relative to simple LOF we subjected LXRα^−^^/−^ mice to the same western diet paradigm as the knock-in mice. Unlike LXRα^W441R/W441R^ mice, weights of knockout mice were comparable with WT littermate controls (Extended Data Fig. 6a–c). While LXRα^−^^/−^ mice exhibited some evidence of hepatotoxicity with modest rises in ALT and fibrosis, this seemed much more modest than the liver injury sustained in LXRα^W441R/W441R^ mice and steatosis was notably preserved (Extended Data Fig. 6d,e,l–n). Free and esterified cholesterol in liver lysate were increased approximately fivefold and sixfold, respectively (Extended Data Fig. 6i,j). Suppression of hepatic triglycerides was not observed in knockout mice and changes in serum triglycerides were more modest than in LXRα^W441R/W441R^ mice (Extended Data Fig. 6f,k,l), suggestive of enhanced repression of lipogenic gene expression by the DN p.W441R variant.

We next performed transcriptomic analysis of livers from LXRα^−^^/−^ and LXRα^W441R/W441R^ mice. In keeping with the hepatotoxic effects of LOF in LXRα generally and its key role in lipid metabolism, we observed directionally concordant changes in inflammatory gene expression and genes related to lipid metabolism in both knockout and knock-in mice (Supplementary Fig. 4b–d and Supplementary Tables 17–20). However, LXRα^W441R/W441R^ mice exhibited more severe dysregulation of canonical LXRα target genes (Supplementary Fig. 4a) and a notable downregulation in genes implicated in lipogenesis and triacylglycerol synthesis (Fig. 4k,l), including *Fasn*, *Srebf1*, *Scd1* and *Dgat2*. Taken together, these findings demonstrate, analogous to other nuclear receptors, that the repressive effects of DN LXRα isoforms result in more severe phenotype than loss of LXRα alone.

### Hepatocyte LXRα is protective in mice fed a western diet

**Fig. 5:**
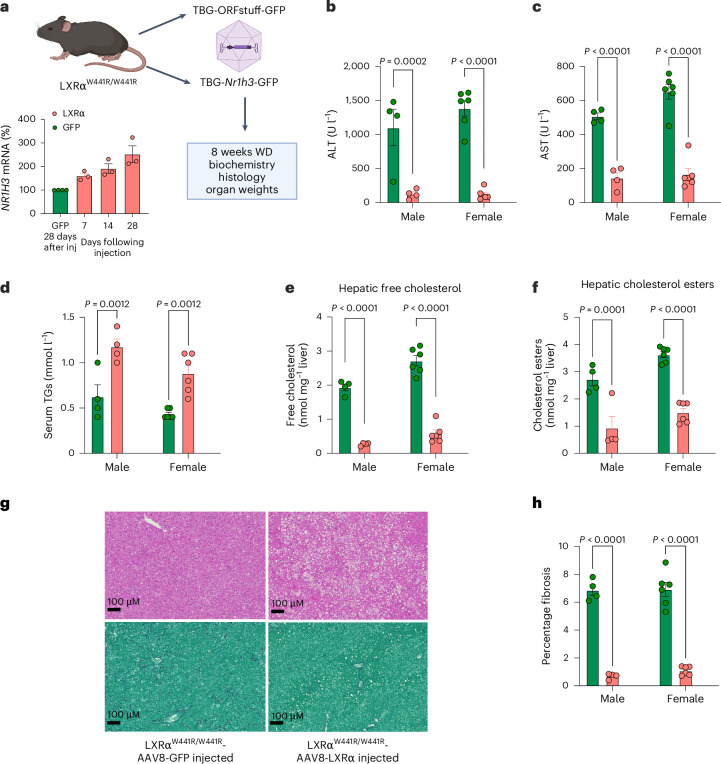
The hepatic injury that results from western diet feeding in LXRα^W441R/W441R^ mice can be rescued by hepatocyte overexpression of WT mouse LXRα. **a**, Schema of study design. Created with biorender.com. Ten male and 12 female 10–12-week-old C57BL/6J LXRα^W441R/W441R^ mice were randomized to receive a tail vein injection with 1 × 10^−^^11^ GC of AAV8 expressing either LXRα and GFP (LXR, salmon bars/symbols) or just GFP (GFP, green bars/symbols) under the control of the hepatocyte-specific TBG promoter (Methods) and 1 week later they were placed on WD. At 4 weeks, this dose resulted in an approximately twofold induction of *Nr1h3* expression in whole-liver lysate (*n*(GFP) = 4 mice, *n*(LXR 7,14 and 28 days) = 3 mice per group). **b**,**c**, Liver enzymes ALT and AST after 8 weeks of WD. **d**, serum TGs after 8 weeks of WD. **e**,**f**, Free and esterified hepatic cholesterol measured using liquid chromatography with mass spectrometry detection. **g**, Representative micrograph of haematoxylin and eosin (H&E) (top) and PSR (bottom)-stained liver sections from each experimental group. **h**, A quantification of fibrotic area derived from HALO analysis of PSR. Throughout, the red bars and symbols represent mice treated with LXRα-expressing virus and green represents mice injected with control GFP-expressing virus. All *P* values are two-sided. All data presented were analysed with a two-way ANOVA with post hoc Holm–Šídak testing. The height of the bars represents the mean ± s.e.m. For data presented in **b**–**f**,**h**, *n*(GFP) = 4 male, 6 female mice, *n*(LXR) = 4 male, 6 female mice. Source data

**Extended Data Fig. 7:**
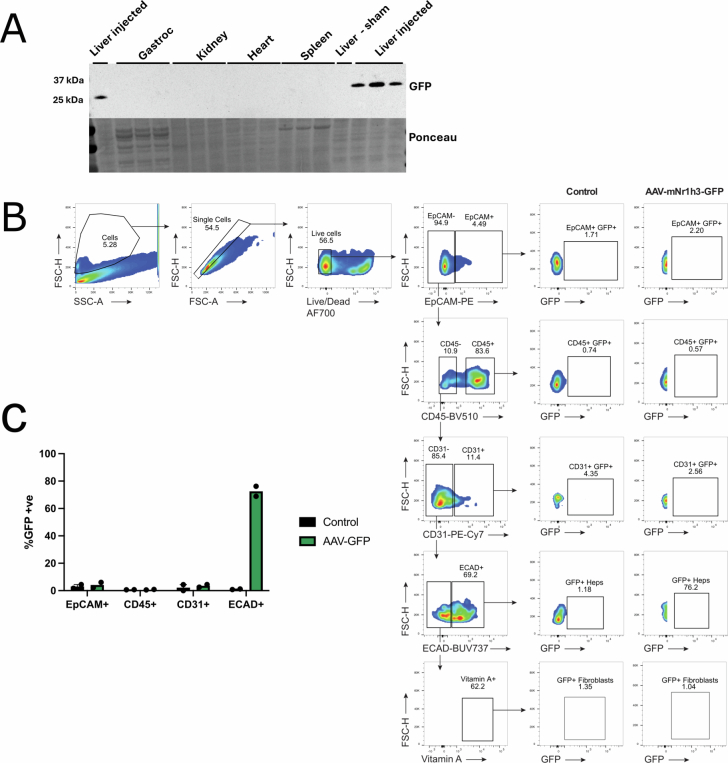
Validation of viral overexpression paradigm. To validate that AAV8-mediated overexpression of wild-type *Nr1h3* was specific we detected the GFP marker gene transcribed from the bicistronic vector which also expressed *Nr1h3* using Western blot (**A**) and Flow Cytometry (**B**). **A:** GFP expression in a panel of selected tissue lysates was analysed by Western blot (N = 3). A sample from a mouse that was subject to a sham injection of AAV8 is shown as a negative control, all other samples are from separate mice injected with AAV8-TBG-*Nr1h3*. **B:** Gating strategy for flow cytometry analysis of a single cell suspension from livers of mice subject to a sham injection or injected with AAV8-TBG-*Nr1h3* (N = 2 mice per group) which expresses marker eGFP gene from the same bicistronic vector as *Nr1h3* (AAV-mNr1h3-GFP). GFP-positive cells are indicative of cellular transduction and is restricted to hepatocytes. **C:** The proportion of GFP-positive cells in each lineage are shown (N = 2). Source data

**Extended Data Fig. 8:**
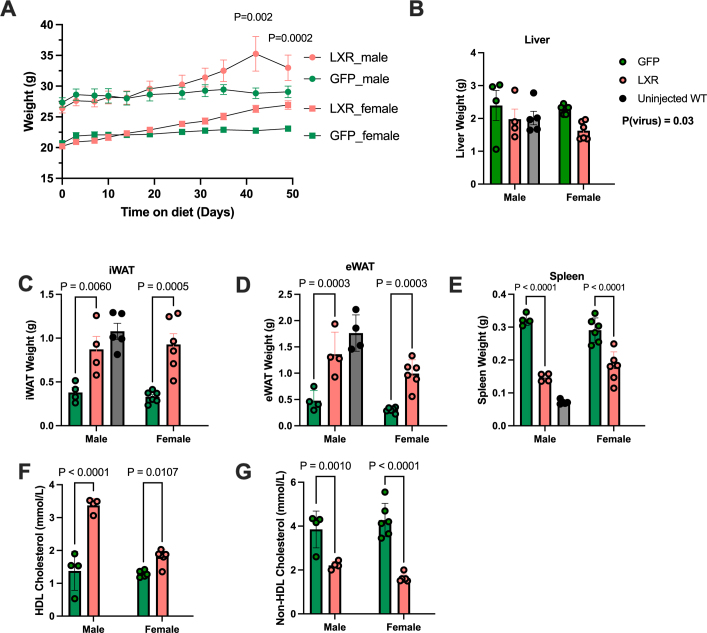
Hepatocyte expression of WT mouse LXRα normalizes bodyweight, organ weights and serum biochemistry in Western diet-fed LXRα^W441R/W441R^ mice. 10 male and 12 female 10–12 week old C57BL/6 J LXRα^W441R/W441R^ mice were randomized to receive a tail vein injection with 1x10^-11^ GC of AAV8 expressing either LXRα and GFP (LXR) or just GFP (GFP) under the control of the hepatocyte-specific Thyroxine binding globulin (TBG) promoter (see methods) and a week later they were placed on western diet. **A:** Body weight curves of mice in each group on a western diet, N(GFP) = 5 male, 6 female mice, N(LXR) = 5 male, 6 female mice. **B-E:** Tissues weights of indicated organs at necropsy **F-G:** Serum cholesterol measurements after 8 weeks on Western diet. Throughout, the red bars and symbols represent mice treated with LXRα expressing virus, green represents mice injected with control GFP-expressing virus, grey indicate non-littermate 10-week old male wild-type mice not injected with virus as a comparison (N = 5). Repeated measures data (bodyweight) was analysed with a mixed-effects model with post hoc between group comparisons undertaken with the Holm-Šídak test with sex included in the model as a co-variate. All other data presented are analysed with a Two-way ANOVA with post hoc Holm-Šídak testing. Height of the bars represents mean ± SEM. For all biochemistry and organ weight data presented N(GFP) = 4 male, 6 female mice, N(LXR) = 4 male, 6 female mice. Source data

**Extended Data Fig. 9:**
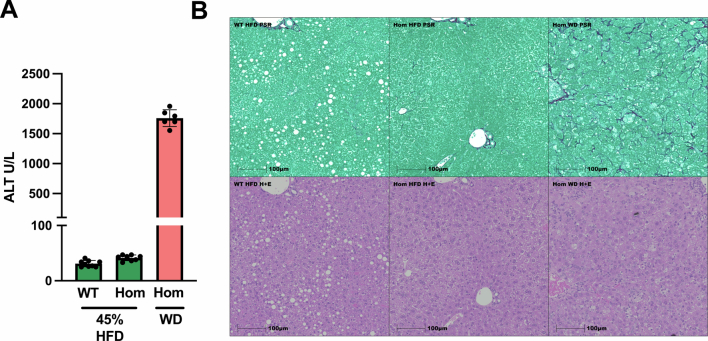
LXRα^W441R/W441R^ mice do not develop fibrotic liver injury on a High Fat, low-cholesterol diet. Age-matched (16–17 weeks) Wild-type (N = 9, WT) or homozygous knock-in mice (N = 8, Hom) fed a 45% High-fat diet (HFD) with low ( ~ 0.02% cholesterol) cholesterol content. An older group of homozygous knock-in mice (2 x 21 weeks, 4 x 31 weeks old) fed a high cholesterol western diet (0.2% cholesterol, WD) for the same period of time, in parallel, were included as a positive control **A:** Serum ALT measured in Error bars represent mean ± SEM after 4 weeks of western diet. **B:** Representative Haematoxylin and Eosin (H + E) and Picosirius red stained (PSR) liver sections from these animals euthanised after 8 weeks of western diet. Source data

In humans and in two different murine models, we observed uncoupling of the lipogenic actions of LXRα from hepatotoxicity. We next sought to determine whether the hepatotoxic effects of LXRα^W441R^ were dependent on hepatocyte LXRα. To do this, we expressed WT LXRα in hepatocytes using AAV8 expressing *Nr1h3* under the control of a thyroxine-binding globulin (TBG) promoter. We achieved a doubling of expression of *Nr1h3* mRNA in liver with LXRα-expressing virus after 28 days (Fig. 5a) without evidence of significant off-target expression (Extended Data Fig. 7a–c). Consistent with the hepatoprotective effects of LXRα being exerted directly in hepatocytes, treatment with an LXRα expressing virus normalized body weight (Extended Data Fig. 8a) and prevented liver injury and hepatic fibrosis (Fig. 5b,c,g,h) despite adverse effects on serum triglycerides (Fig. 5d) and restoration of hepatic steatosis (Fig. 5g). Notably, adipose tissue weights were normalized and spleen size was partially normalized by hepatocyte LXRα expression, suggesting that reduced adiposity and exacerbation of splenomegaly are secondary consequences of liver injury rather than a primary effect of impaired LXR signalling (Extended Data Fig. 8c–e). Consistent with liver injury being dependent on hepatic cholesterol accumulation, hepatic cholesterol levels were markedly reduced by hepatocyte LXRα expression (Fig. 5e,f) and LXRα^W441R/W441R^ mice did not develop significant liver injury when fed a high-fat, high-sucrose diet with a low (~0.02%) cholesterol content (Extended Data Fig. 9).

### Liver injury in LXRα^W441R/W441R^ mice is hepatocellular in nature

**Extended Data Fig. 10:**
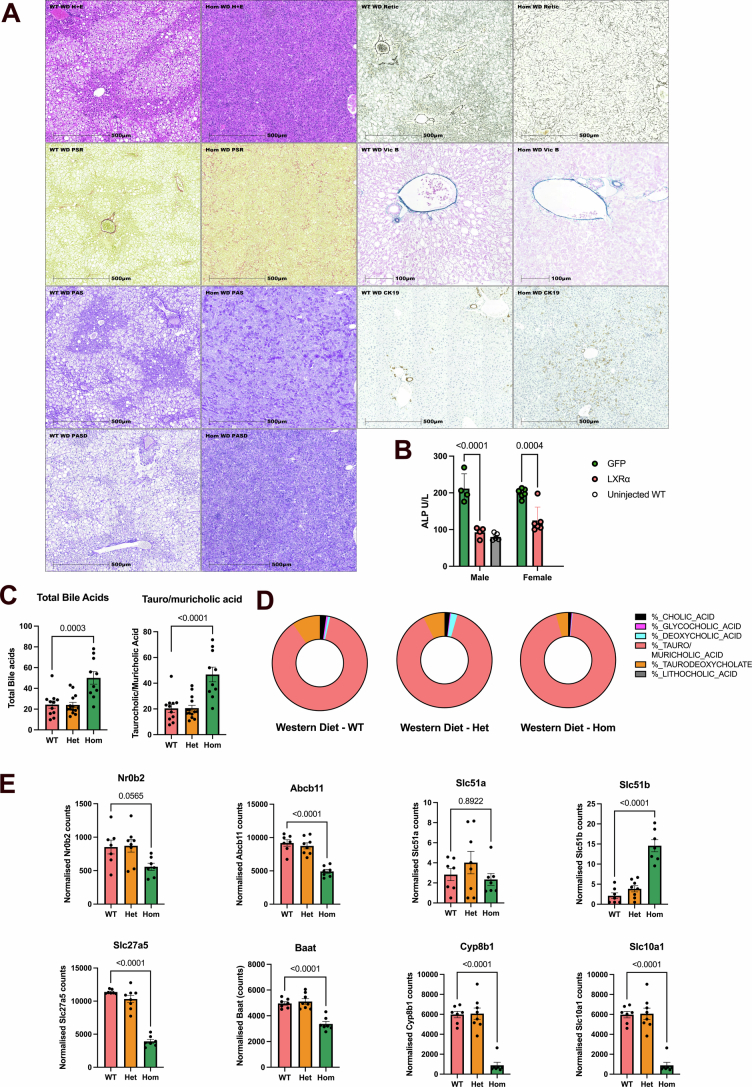
Histopathological evaluation of liver injury in LXRα^W441R/W441R^ mice and bile acid measurements. **A:** Detailed histopathological assessment of LXRα^W441R/W441R^ mice livers. Liver sections from 3 randomly selected Wild-type (WT) and 3 randomly selected homozygous knock-in mice (HOM) fed a western diet (WD) for 8 weeks were subject to a battery of histopathological stains, representative images are shown. H + E: Haematoxylin and eosin, PSR: Picrosirius red staining, PAS: Periodic Acid Schiff, PASD: Periodic Acid Schiff with diastase, Retic: Reticulin, Vic B: Victoria Blue, CK19: Immunohistochemistry for cytokeratin-19. **B:** Circulating ALP was measured in residual serum from Homozygous knock-in mice treated with either a GFP control AAV or an AAV expressing LXRα under control of a liver specific promoter as described in Fig. 5, or age-matched Wild-type mice, after 8 weeks of western diet feeding (N = 4 males and 6 females per group) **C, D:** Bile acids in liver lysate were determined by LC-MS in Wild-type, Heterozygous and Homozygous Knock-in mice (LXRα^W441R/W441R^), quantitation of total bile acids and the predominant group of bile acids (Taurocholic and muricholic acid, which cannot be differentiated in our method – Tauro/murocholic acid) N(WT) = 11, N(Het)=14, N(Hom)=10 (**B**), with relative proportions of bile acid in each genotype shown in (**C**) **E:** Expression of FXR-target genes from bulk RNA-seq data of livers from Wild-type, Heterozygous and Homozygous Knock-in mice (LXRα^W441R/W441R^) are presented as an index of bile acid-dependent signalling in these mice. N(WT) = 7, N(Het)=8, N(Hom)=7. All error bars represent mean ± SEM. Hypothesis testing was conducted by One-way Anova with post hoc Holm-Sidak test. P values are two-sided P values. Source data

To provide additional insight into the aetiology of liver injury in the LXRα^W441R/W441R^ mice, we conducted additional detailed histopathological and biochemical characterization of these animals (Extended Data Fig. 10). Periodic acid Schiff (PAS)/periodic acid Schiff diastase (PASD) staining demonstrated PAS-positive, PASD-negative hepatocyte cytoplasm, consistent with hepatic glycogen, rather than microvesicular steatosis (Extended Data Fig. 10a). The pattern of inflammatory infiltrate was diffuse, present throughout the hepatic parenchyma; no cholestasis was observed and each portal tract was associated with at least one bile duct. Victoria blue staining showed no copper accumulation (Extended Data Fig. 10a) and a biomarker of cholestatic liver injury, serum alkaline phosphatase (ALP), measured in residual serum from western-diet-fed LXRα^W441R/W441R^ mice was only modestly elevated compared with littermates rescued with AAV-mediated expression of WT LXRα and WT control mice (Extended Data Fig. 10b), whereas ALT, primarily a biomarker of hepatocellular pathology, was markedly elevated (Fig. 5b). These findings suggest a hepatitic rather than cholangiopathic pattern of injury.

### Liver bile-acid profiles in LXRα^W441R/W441R^ mice

While the liver injury seems unlikely to be driven solely by a primary cholestatic pathology, alterations in bile-acid profile and bile-acid signalling play a role in the pathogenesis of pathologies typically viewed as hepatitic in nature, such as MASLD^43–45^. By using mass spectrometry to profile hepatic bile acids in LXRα^W441R/W441R^ mice, we observed an increase in total bile acids, driven primarily by increases in taurocholic/muricholic acid (these species cannot be distinguished in our method) (Extended Data Fig. 10c,d). Bile-acid accumulation could be directly hepatotoxic or fibrogenic by activation of stellate cells^46^ or could be adaptive via activation of FXR signalling and its pleiotropic actions on hepatocyte metabolism and immune cells^45^. To evaluate FXR-dependent signalling in our model we queried our RNA-seq data for FXR-target gene expression. This demonstrated discordant effects on FXR-target genes with upregulation of the hepatocyte taurocholate exporter *Slc51b* and repression of the uptake transporter *Slc10a1* (ref. ^47^) and driver of cholic acid synthesis *Cyp8b1*, which are consistent with enhanced FXR-dependent signalling in LXRα^W441R/W441R^ mice (Extended Data Fig. 10e). In contrast, the key FXR-effector gene *Nrob2* was not induced, while other positively regulated FXR-target genes *Baat*, *Abcb11* and *Slc27a5* were actually repressed. Thus, LXRα^W441R/W441R^ mice have increased hepatic bile acids and an altered bile-acid profile with apparently complex effects on canonical target genes of bile-acid signalling.

### Cholesterol crystal burden and severity of liver injury

**Fig. 6:**
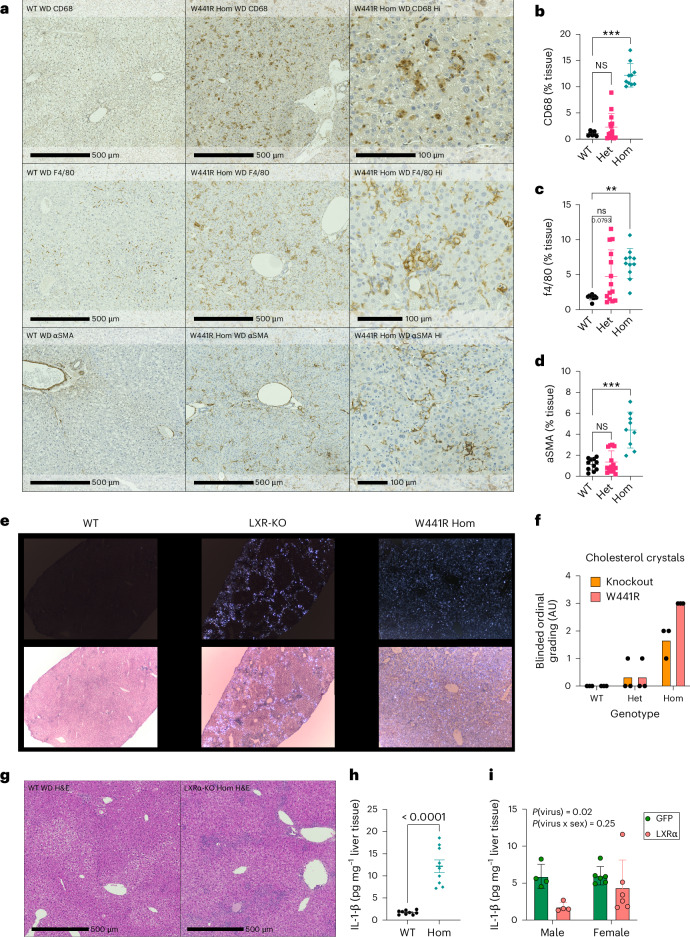
Enhanced inflammation and cholesterol crystallization in LXRα^W441R/W441R^ mice. **a**, Immunohistochemical assessment of macrophage abundance (CD68, F4/80) and stellate cell activation (αSMA), in WD-fed WT and homozygous knock-in mice (W441R Hom). **b**–**d**, Quantitation of immunohistochemical staining. Statistical analysis was conducted with Kruskal–Wallis test with post hoc Dunn’s test. *n*(WT) = 8, *n*(Het) = 14, *n*(Hom) = 10 (**b**); *n*(WT) = 9, *n*(Het) = 14, *n*(Hom) = 10 (**c**); *n*(WT) = 11, *n*(Het) = 14, *n*(Hom) = 9 (**d**). **e**, Representative low-power images from frozen sections of livers from WT, LXRα knockout mice (LXR-KO) and homozygous knock-in (W441R Hom) mice viewed under a polarized light demonstrating presence of cholesterol crystals in the LXRα KO mice, which seems to spare steatotic regions and homozygous knock-in livers where cholesterol crystals are diffusely abundant. **f**, Ordinal grading of cholesterol crystal content conducted by a histopathologist blinded to genotype. 0, absent; 1, minimal; 2, marked but patchy or moderate and diffuse; 3, marked and diffuse. *n* = 3 animals per group. **g**, H&E staining of LXRα KO and WT livers demonstrating patchy inflammatory infiltrate mirroring the pattern of cholesterol crystal accumulation. **h**,**i**, IL-1-β measured in liver lysates from WT and homozygous knock-in mice (Hom) fed WD for 8 weeks, *n* = 9 per group (**h**) or homozygous knock-in mice fed WD for 8 weeks treated with either a control virus (GFP) or a virus expressing LXRα in hepatocytes, which rescues liver injury (Fig. 5), *n* = 6 males and four females per group. Analysis in **h** was conducted by Mann–Whitney *U*-test and by two-way ANOVA in **i**. The height of the bars represent mean ± s.d. All *P* values are two-sided. ***P = 0.0002 WT versus Hom (**b**); **P = 0.001 WT versus Hom (**c**); ***P = 0.0006, WT versus Hom, NS, *P* > 0.05 (**d**); *P* < 0.0001 (**h**). Source data

Having established that impaired hepatocyte LXRα signalling was hepatotoxic and associated with histological evidence of liver inflammation, we sought to explore this in greater detail and undertook immunohistochemical assessment of inflammation in LXRα^W441R/W441R^ mice. We observed allele-dependent increase in Kupfer cell markers CD68 and F4/80 in the liver (Fig. 6a–c) and more modest increases in the T cell marker CD3 Extended Data Fig. 5h). We also observed increased alpha-smooth muscle actin (αSMA) immunoreactivity consistent with stellate cell activation (Fig. 6a,d). Inflammatory reactions to cholesterol-laden hepatocytes have been noted to centre on lipid droplets, forming crown-like structures^32,48^. Absence of steatosis means this histopathological hallmark was notably absent in our model, but we did observe inflammatory cells focused on hepatocytes (Fig. 6a), and xanthogranulomata, suggestive of an immune reaction to cholesterol-laden hepatocytes. When free cholesterol concentrations rise, cholesterol can crystalize and is one purported mechanism of cholesterol-mediated hepatotoxicity^25,26,32,49^. We conducted a blinded assessment of frozen sections by cross-polarized microscopy. Cholesterol crystals were not observed in WT mice. They did occur in LXRα knockout mice, but were concentrated in the periportal region in areas that were devoid of steatosis. In contrast, cholesterol crystal accumulation was severe and diffuse in LXRα^W441R/W441R^ mice (Fig. 6e,f). There was a correlation between the pattern of inflammatory infiltrate in our respective models and the pattern of cholesterol crystals, with inflammatory cells noted throughout the liver parenchyma in LXRα^W441R/W441R^ mice, whereas this seemed to be predominantly located in periportal regions in LXRα knockout mice where steatosis was less notable, sparing heavily steatotic pericentral hepatocytes (Fig. 6e–g). Cholesterol crystals are known to drive inflammation via activation of the NLRP3 inflammasome^50^. Consistent with this, we observed marked increases in the key effector cytokine of the NLRP3 inflammasome Il-1-β in LXRα^W441R/W441R^ mice and this could be prevented by expression of LXRα in hepatocytes (Fig. 6h,i). Thus, our data are consistent with a model whereby hepatocyte cholesterol accumulation results in cholesterol crystal formation, which activates an immune response resulting in inflammation and subsequent fibrosis.

## Discussion

Previous studies have established that LXRα plays key roles in sensing cholesterol metabolites to regulate lipid synthesis and secretion in liver and some other tissues. However, questions remain regarding its role in human biology. By examining the impact of rare, damaging mutations in LXRα on human phenotypes and in and mice expressing such mutants we have gained insights onto the role of this nuclear receptor in the control of human cardiometabolic and hepatic health.

Elevation of HDL cholesterol was the strongest association in our analysis of damaging mutations in LXRα in UKBB, confirming that the net effect of endogenous LXRα agonism in humans is to suppress HDL cholesterol. In contrast, synthetic LXR agonists elevate HDL cholesterol in mice^11,12,51^. It is likely that these species differences are due to the ability of LXRα to induce CETP^52^, an enzyme that exchanges triglyceride in triglyceride rich lipoproteins for cholesterol esters from HDL, thus lowering HDL cholesterol, but is absent in mice^11,12,51^. The lipoprotein profile seen in humans carrying damaging mutations in LXRα is highly suggestive of CETP deficiency, with triglycerides carried in small and medium HDL the most downregulated and cholesterol ester in large and very large HDL the most upregulated of all lipids tested^12,40^. Previous studies have reported a lower level of HDL cholesterol in humans with specific LOF mutations in LXRα^53,54^, through the study of a broad range of mutations with varying degrees of functional impairment, our study reveals a dose–response relationship between LXRα transactivation capacity and circulating HDL cholesterol.

LXRα mutations have not previously been reported to be associated with markers of hepatic injury. We found that damaging mutations in human LXRα are associated with elevations in circulating levels of liver enzymes despite reductions in circulating VLDL triglycerides and a trend to reduction in liver fat. As LXRα is such a key regulator of cellular cholesterol levels in the liver^27^, these findings suggest that dysregulation of hepatic cholesterol might be hepatotoxic in humans. Observational studies have shown an association between hepatic free cholesterol and presence of steatohepatitis in MASLD^55^, but these studies are susceptible to confounding and reverse causation. Genetic variants that impair VLDL secretion from the liver, including LOF mutations in *APOB* and *TM6SF2* increase risk of liver disease^18^ and presumably increase hepatic cholesterol, as has been shown in some rodent models^56^. However, failure to secrete VLDL particles will also cause accumulation of hepatic triglycerides, so the effect of cholesterol accumulation due to these genetic variants cannot be dissociated from that of triglycerides. By dissociating hepatic cholesterol accumulation from hepatic lipogenesis, damaging mutations in LXRα provide a unique genetic instrument with which to interrogate the hepatotoxic effects of hepatic cholesterol accumulation in humans. While previous studies have studied rare genetic variation and ALT as a proxy for liver disease^18^, these have not reported associations with *NR1H3*, likely due to analytical choices regarding the classification of missense variants. Our study circumvents these limitations in bioinformatic annotation by leveraging high quality experimental data on functional effects of missense variants in *NR1H3*.

LXRα^W441R/W441R^ mice exhibited a striking fibrotic liver injury after 8 weeks exposure to a modest dietary cholesterol challenge, despite the absence of discernible steatosis and markedly reduced hepatic triglycerides. While liver injury was observed in LXRα^−/−^ mice over 20 years ago^27^ this occurred after several weeks on a diet with ten-times more cholesterol. Moreover, in that study, liver triglycerides were only modestly decreased after 90 days on a high-cholesterol diet and there was histological evidence of marked steatosis^27^. We undertook a direct comparison of LXRα^−^^/−^ and LXRα^W441R/W441R^ mice on a western diet. While fibrotic liver injury was observed in knockout mice, it was much more modest in comparison with LXRα^W441R/W441R^ mice and hepatic triglyceride levels were comparable with WT mice. Consistent with these findings, lipogenic gene expression was suppressed to a far greater extent in LXRα^W441R/W441R^ than LXRα^−/−^ mice.

Our hepatocyte-specific rescue experiments confirm that the liver damage resulting from this mutation originates in the hepatocyte itself. This likely occurs as a result of free cholesterol accumulation and subsequent formation of cholesterol crystals, which act as a nidus for the marked inflammatory response observed. It is less clear whether the notable changes in hepatic triglycerides and steatosis are relevant. While hepatic cholesterol crystal accumulation and inflammation was diffuse and severe in LXRα^W441R/W441R^ mice, in LXRα^−/−^ mice it was limited to the periportal regions where steatosis was more limited. This raises the suggestion that the lipogenic effects of LXRα could be protective in the context of cholesterol excess^25^. Indeed, it has been demonstrated that triglycerides facilitate storage of cholesterol esters in lipid droplets via a physicochemical mechanism^57^. One plausible hypothesis is that lipogenesis facilitates cholesterol storage in lipid droplets, which protects cell membranes from toxic cholesterol accumulation and potentially ‘walls off’ any cholesterol crystals that may form in the cytoplasm. While we have demonstrated that liver injury in LXRα^W441R/W441R^ mice can be prevented by viral expression of WT LXRα this does not exclude a role for impairment in LXRα in other hepatic cells in exacerbating liver injury that ensues when cholesterol accumulates due to impaired hepatocyte LXRα^58,59^.

There are some direct translational implications of our work. Most notably, we have shown in humans and in two different mouse models that genetic impairment of LXRα is associated with hepatotoxicity. These findings caution against the use of inverse LXR agonists, agents that are currently in clinical development for MASLD and dyslipidaemia^23^. Unfortunately, due to the limited number of cases of liver disease in the UKBB and the rarity of damaging mutations in LXRα, we were unable to conclusively demonstrate an effect of haploinsufficiency for LXRα on ‘hard’ liver disease end points. We did find an increased risk of alcohol-related liver disease in carriers of damaging LXRα mutations, but this was based on a small number of affected carriers. Future studies conducted in disease cohorts of large sample size will be required to definitively assess the effect of LXRα haploinsufficiency on alcohol-related liver disease and determine whether LXRα modulates risk of liver cirrhosis in response to other hepatotoxic agents.

In summary, using studies in mice and humans, we demonstrate that damaging mutations in LXRα are hepatotoxic, at least in part by increasing hepatocyte cholesterol levels. Our work provides evidence that intact hepatic cholesterol sensing is important in human liver health.

## Methods

### Ethical regulations for studies in humans

The UKBB data have approval from the North West Multi-centre Research Ethics Committee as a research tissue bank. The Fenland study was approved by the Cambridge local research ethics committee (ref. 04/Q0108/19) and all participants provided written informed consent. All analyses reported here were conducted in accordance with relevant ethical guidelines.

### Ethical regulations for studies in animals

In the United Kingdom, all mouse studies were performed in accordance with UK Home Office Legislation regulated under the Animals (Scientific Procedures) Act 1986 Amendment, Regulations 2012, following ethical review by the University of Cambridge Animal Welfare and Ethical Review Body or that of Newcastle University. At the University of California, Los Angeles (UCLA), all animal experiments were approved by the UCLA Institutional Animal Care and Research Advisory Committee. All analyses reported here were conducted in accordance with relevant ethical guidelines.

### Functional classification of rare variants in the ligand-binding domain of LXRα

#### Variant identification and prioritization in UK Biobank

We inspected the exomes of 454,756 UKBB participants to identify and prioritize rare variants for experimental characterization. Processing, quality control (QC) and annotation of sequencing data was undertaken as previously described, with all annotations undertaken with reference to the MANE select transcript (ENST00000441012)^60^. We selected missense variants in the ligand-binding domain of LXRα with a MAF < 0.001 and either a CADD score >23 or REVEL > 0.7 for experimental characterization. These are arbitrary thresholds based on the distribution of REVEL and CADD scores in damaging mutations identified in a pilot study, as well as the number of variants meeting these criteria. We also characterized a small number of PTVs occurring at the C terminus of the protein that we considered likely to act in a dominant negative manner.

#### Variant identification in the Fenland study

To discover carriers of damaging mutations in the ligand-binding domain of LXRα in the Fenland study (a population cohort study, previously described in detail^61^), we sequenced the coding region of *NR1H3* using a pooled-sequencing approach as previously described^62^. Participants who carried a variant of interest needed to be identified from the DNA pool of 20 participants. This was achieved by Sanger sequencing all the participant DNAs from the pool individually. Variants were filtered using the criteria described above for the UKBB, resulting in the identification of two additional variants that were experimentally characterized.

For cloning and site-directed mutagenesis, the *NR1H3* cDNA construct (NM_005693) from pDNA3.1-hLXRα was cloned into a pcDNA3.1(+) vector (V79020, Thermo Fisher). This plasmid was used to generate mutants throughout the study. For yeast two-hybrid assays, we used pCMX-VP16-LXRα for mutant generation^63^. Site-directed mutagenesis of *NR1H3* was performed using QuikChange Lightning Site-Directed Mutagenesis kit (210519, Agilent Technologies) according to the manufacturer’s protocols. All constructs were verified with Sanger sequencing and DNA was extracted using a Plasmid Maxi kit (12163, QIAGEN). To characterize the functional consequences of LXRα mutants we performed assays in transiently transfected HEK293.

#### Cell culture

HEK293 (XX female) cells and HepG2 cells were obtained from the laboratory stock (originally purchased from The European Collection of Authenticated Cell Cultures), cultured in high-glucose Dulbecco’s modified Eagle’s medium (41965, Thermo Fisher) and supplemented with 10% fetal bovine serum (10270, Thermo Fisher, South America origin), 1% 100× GlutaMAX (Thermo Fisher, 35050) and 100 U ml^−1^ penicillin and 100 mg ml^−1^ streptomycin (P0781, Sigma-Aldrich). Cells were incubated at 37 °C in humidified air containing 5% CO_2_. Transfections were performed in HEK293 cells using Lipofectamine 3000 Transfection Reagent (L3000015, Thermo Fisher) and serum-free Opti-MEM I medium (31985, Thermo Fisher) according to the manufacturer’s protocols.

#### Dose–response LXRα transactivation assays

To assess the effect of LXRα variants, WT and different LXRα mutants were transiently expressed in HEK293 cells and the ligand-induced transcriptional activity was measured using a Dual-Glo Luciferase Assay System (E2940, Promega) according to manufacturer’s protocols. In brief, 30,000 live cells were seeded in white 96-well poly-d-lysine-coated plates. After 24 h, cells were transfected with 30 ng per well of LXRRE luciferase plasmid^64^, 30 ng per well of pRL-TK Renilla luciferase transfection control plasmid (2241, Promega) and 40 ng per well of pcDNA3.1-LXRα or mutant plasmid. After 4 h from transfection, the cell medium was replaced by 75 µl medium with 0–1,000 nM of T0901317 ligand. Then, 24 h post-transfection, firefly and Renilla luciferase activities were measured subsequently using a Spark 10M microplate reader (Tecan). For normalization, firefly values were divided by Renilla values and presented as %WT for each independent experiment. Each mutant was tested in a minimum of three independent experiments, resulting in a total of 25 independent experiments conducted across seven individual batches. A two-way repeated-measures analysis of variance (ANOVA) was computed within each experimental batch using the afex package. Unadjusted post hoc comparisons to the WT condition were conducted in emmeans and resultant *P* values were then adjusted using the Benjamini–Hochberg method to control the false discovery rate (FDR).

#### Co-expression LXRα transactivation assays for DN

Assays performed as above, with the following changes. Only 10 ng per well of each of pRL-TK Renilla luciferase plasmid and LXRRE luciferase plasmid were transfected per well together with 40 ng per well of pcDNA3.1-LXRα WT vector and 40 ng per well of pcDNA3.1-LXRα mutant vector. After 4 h, the medium was changed only to basal (0 nM T0901317) or maximum (1,000 nM) ligand concentration. Firefly/Renilla values were normalized to % EV + WT, presented as mean ± s.d. and compared by a one-sample *t*-test.

#### Mammalian two-hybrid assays

As described above, cells were plated at 30,000 live cells per well in a pre-coated 96-well plate. After 4 h, the wells were transfected with 25 ng UAS-TK-Luc reporter construct, 13 ng of pCMV-VP16 (EV) or pCMV-LXRα-VP16 and 13 ng of GAL4 co-factor vector and 13 ng pRL-TK (Promega, 2241) using Lipofectamine 3000. In the case of co-activator studies, the Gal4-SRC1 (aa 570-780)^65^ construct was used, while Gal4-NCoR (ID 1 + 2)^65^ was utilized for the study of co-repressor protein–protein interaction. The culture medium was replaced with fresh medium containing increasing doses of the LXRα ligand T0901317 at 4 h following transfection and incubated for a further 20 h before luciferase activity was measured as above. Mutants were tested in three separate batches with three independent experiments per batch. Results were normalized (firefly/Renilla) and then presented as a percentage of average response in the 1,000 nM condition for co-activator (SRC1) association assays and percentage of 1 nM condition for co-repressor association (NCoR) within each batch. Sigmoidal dose–response curves with variable slope (three-parameter logistic regression) were fitted and plotted using Prism (GraphPad).

#### Statistical analyses of mammalian two-hybrid assay experiments

For statistical analyses of the effects of LOF and DN mutations on co-activator and co-repressor association as an aggregated class, we used mixed-effects models with normalized luciferase activity as the outcome variable and mutant class and agonist dose as fixed effects (with interactions) with individual mutant and experimental replicates included in the model as random intercepts, implemented in the package LmerTest. Mutant activity was log-transformed before analysis. As only one GOF mutant was tested in a single batch of three replicates, it was assessed independently using a mixed-effects model with experimental replicate included as a random intercept with normalized luciferase activity log-transformed before analysis. Post hoc testing using the emmeans package was conducted between the WT group and LOF, DN and GOF groups at each dose of agonist included in the experiment, using Dunnett’s test.

### Human studies

#### Burden testing in UK Biobank

##### Primary analysis

Criteria for variant masks are detailed in Supplementary Table 2. All criteria for classification were defined a priori before association testing. Variant lists for each mask were then used as input to the ‘collapsevariants’ applet from the MRC-EPID WES pipeline^60^ for use in downstream association testing.

For our primary analysis we pre-specified a set of 16 phenotypes (Supplementary Table 4) relevant to cardiometabolic health and previously described functions of LXRα from animal and cellular studies. Details of relevant UKBB fields and phenotype processing are described in Supplementary Table 4.

Association testing was conducted using the MRC-EPID WES pipeline on the UKBB RAP using the ‘extract’ function on the applet ‘MRC-EPID-runassociationtesting’ to test association with each variant mask against each trait of interest, as described previously^60^. In brief, this applet runs a generalized linear STAAR^66^ model. We restricted our analyses to UKBB participants of European ancestry. Age, age squared, genetic sex, whole-exome sequencing batch and the first ten genetic principal components as defined by Bycroft et al.^67^, were included as covariates for all phenotypes. Lipid and glycaemic traits were adjusted for use of lipid-lowering, blood pressure or diabetes medications as detailed in UKBB fields 6153 and 6177 (Supplementary Table 4).

To determine whether DN mutations and PTVs had effects similar to ‘simple’ LOF missense mutations, we undertook a linear model to regress the effect of ‘simple’ LOF missense mutations for each test phenotype against PTVs and DN variants using the inverse variance of the effect estimate as weights in the linear model. Finding similar effects of LOF, DN and PTVs, we aggregated these into a single mask of all damaging variants to maximize statistical power. We considered associations significant in our discovery analysis if the STAAR Burden *P* value was less than a Bonferroni-corrected threshold of *P* < 3.9 × 10^−4^, accounting for 128 mask × phenotype association tests.

Description of additional statistical genetics analyses are available in the online supplement.

### Mouse studies

#### Animal housing

Studies were carried out at three sites: the University of Cambridge, Newcastle University and UCLA. Mice were maintained on a 12-h light–dark cycle (lights on 07:00 to 19:00) in a temperature-controlled (22 °C) facility, with ad libitum access to food and water. At UCLA, LXRα knockout (LXRα ^−/−^) mice originally on a mixed Sv129/C57Bl/6 were obtained from D. Mangelsdorf and backcrossed more than ten generations to the C57BL/6 background. LXRα-deficient mice (Hom), heterozygous (Het) and WT mice were maintained in a temperature-controlled room and a 12-h light–dark cycle. Food and water were available ad libitum. Animals at both institutes were maintained on RM3(E) Expanded Chow (Special Diets Services). Where specified, mice were fed a western diet ad libitum (TD.88137, 42% kcal from fat, 32% sucrose by weight and 0.2% cholesterol, Envigo) or a control diet (TD.05230, 12.6% kcal from fat, 32% sucrose by weight and 0.05% cholesterol, Envigo).

#### Generation of LXRα^W441R^ mice

In brief, one-cell-stage C57BL/6J (Janvier Labs) embryos were injected with 50 ng μl^−1^ 32fw sgRNA (IDT), 100 ng μl^−1^ TriLink CleanCap Cas9 mRNA (L-7606) and 50 ng μl^−1^ Alt-R 32fw donor top strand 123 bp ssODN (IDT). Injected embryos were briefly cultured and the viable embryos were transferred the same day into pseudo-pregnant F1 (C56BL/6J/CBA) recipients. The F0 founder, carrying the desired mutation, was crossed with WT C57BL/6J (Charles River Laboratories) mice to segregate the mutations introduced and to create F1 founder mice. Mutations were confirmed with Sanger sequencing. Founder F1 mice were crossed one more time with WT C57BL/6J mice before establishing the mutant mouse colony. Mutations were confirmed with Sanger sequencing.

#### Genotyping strategy for LXRα^W441R^ mice

Mice were genotyped using standard PCR using primers 538_Nr1h3_amplicon_709_F1 (CCCTACAGTGGATGAGAGAGGT) and 539_Nr1h3_amplicon_709_R1 (CAACGTTAGTAGATCCCAACTGC). PCR products were digested with BglII and separated on 1.5% agarose gel. The BglII digest produces two bands (497 bp and 212b) for the WT mice and three bands (709 bp, 497 bp and 212 bp) for the heterozygous mice. BglII will not cut the PCR product for the homozygous mice as the mutation destroys the restriction enzyme site.

#### Mouse study 1: western diet and control diet study of LXRα^W441R^ mice

Two experimental cohorts of male homozygous (LXRα^W441R/W441R^), heterozygous (LXRα^+/W441R^) and WT (LXRα^+/+^) mice were generated by het × het breeding pairs. Both cohorts were weighed weekly between 4 and 16 weeks of age and had their body composition (lean and fat mass) assessed by ECHO MRI M113 mouse system (Echo Medical Systems) at 8 and 16 weeks of age. At ~8 weeks of age, the first cohort of mice was transferred from standard chow to a western diet. At the same age, the second cohort was transferred to a control diet. During the experiment, one mouse died unexpectedly, as such, three mice of each genotype of the western diet cohort were killed early at 14 weeks of age and pathologically assessed to exclude genotype-dependent pathology that would jeopardize animal welfare for the remainder of the experiment. This assessment revealed reduced fat mass, splenomegaly and a hardened liver, consistent with fibrosis, as described in the main manuscript for the whole cohort, without gross evidence of other pathology. At ~16 weeks of age, all remaining mice were killed by CO_2_ asphyxiation and blood was isolated using cardiac puncture. Inguinal and gonadal white adipose tissues, interscapular brown adipose tissue and spleen were weighed before splitting in two, with one part fresh frozen in liquid nitrogen and the second fixed in 10% formalin. The left kidney, heart and pancreas were also isolated and fresh frozen. Blood samples were centrifuged at 4 °C and the separated plasma was stored at −70 °C until used for analysis. The cohort assignment to either the control or western diet was arbitrary but not formally randomized.

#### Mouse study 2: western diet study of LXRα^−/−^ mice

Homozygous (LXRα^−/−^), heterozygous (LXRα^+/−^) and WT (LXRα^+/+^) mice were generated by het × het breeding pairs. At 8 weeks of age, the first cohort of mice was transferred from standard chow to western diet. At 16 weeks of age, the mice were weighed and their body composition was assessed by ECHO MRI (3-in-1). The mice were then killed and bled by cardiac puncture. Serum, liver, inguinal and gonadal white adipose were weighed and fresh frozen in liquid nitrogen and/or fixed in 10% formalin.

#### Mouse study 3: adenoviral rescue experiment in LXRα^W441R^ mice

To determine the dependency of the liver phenotype in LXRα^W441R/W441R^ mice, we overexpressed mouse WT LXRα (encoded by *Nr1h3*) using AAV8-TBG-dependent expression of *Nr1h3*. In brief, 10–12-week-old, male and female LXRα^W441R/W441R^ mice were injected via the tail vein with 1 × 10^−11^ genomic copies of commercially produced (VectorBuilder) adeno-associated virus (serotype 8, AAV8) containing a vector expressing an eGFP marker gene and either, WT mouse LXRα (AAV8–TBG–Nr1h3) or an ORF-stuffer (AAV8–TBG–GFP), from a bicistronic vector downstream of the liver specific promoter TBG. After 1 week, they were placed on a western diet and were killed after 8 weeks on a western diet for blood and tissue collection. The specificity of this approach was confirmed by (1) western blotting for green fluorescent protein (GFP) in the liver (Cell Signalling, 2596S) and other tissue lysates; and (2) flow cytometry to identify GFP-positive cells, described below. Assignment to either virus was conducted by randomization stratified for sex.

#### Mouse study 4: high-fat, low-cholesterol diet challenge in LXRα^W441R^ mice

Age-matched, 16–17-week-old male and female WT and homozygous LXRα^W441R^ mice were fed a high-fat (45%), high-sucrose (17%), low-cholesterol (0.02%) diet (Research Diets, D12451) for 8 weeks. An older cohort of homozygous LXRα^W441R^ mice were fed a western diet for a total of 8 weeks (Envigo TD.88137) as a positive-control group. After 4 weeks on the study diet, blood was obtained from the pedal vein, processed for serum and ALT was analysed as described below. After 8 weeks on the study diet, mice were killed and the livers were processed for histopathological analysis as described below.

#### Mouse serum biochemistry

Mouse blood was collected in serum separator tubes following CO_2_ asphyxiation using cardiac puncture, clotted for 10 min at room temperature, then spun at 8,000*g*. The serum was then collected and snap frozen on dry ice or in liquid nitrogen. Serum triglycerides, ALP, transaminases, lipoproteins and cholesterol were measured on a Dimension EXL analyser (Siemens Healthcare) or PerkinElmer DELFIA using reagents and calibrators (Siemens).

#### RNA extraction

As fibrotic livers were not properly homogenized using ceramic beads, homogenization was performed on all liver samples using metal beads, Trizol or Direct-zol (Zymo) and VelociRuptor V2 Microtube Homogenizer (SLS Lab Pro). Total RNAs was extracted from the mouse livers or HepG2 cells with RNeasy kit (QIAGEN) or Direct-zol RNA Miniprep kit (Zymo Research), respectively.

#### Quantitative PCR with reverse transcription

For quantitative PCR with reverse transcription (qRT–PCR) analysis, 0.5–1 μg HepG2 RNA was reverse transcribed using M-MLV Reverse Transcriptase (Promega) according to the manufacturer’s protocol. qPCR reactions were prepared using TaqMan probes and TaqMan Universal PCR Master Mix (Thermo Fisher) according to the protocol and were quantified using the default TaqMAN program on ABI QuantStudio 5.

#### RNA sequencing and bioinformatic analyses

RNA quality was confirmed using the Agilent 2100 Bioanalyzer or Agilent TapeStation system. For HepG2 cells, RNA-seq libraries were prepared using the TruSeq RNA CD Indexes and the Illumina Standard mRNA Prep kit according to Illumina protocol. Multiplexed libraries were validated using the Agilent TapeStation system, normalized and pooled for sequencing. High-throughput sequencing was performed on NovaSeq 6000 (Illumina) at PE50 at the Cancer Research UK (CRUK) Cambridge Institute Genomics Core Facility. Image analysis and base calling were performed with Illumina CASAVA v.1.8.2. For mouse livers, RNA was processed by Novogene Co. In brief, stranded messenger RNA libraries were constructed by using Novogene NGS Stranded RNA Library Prep Set (PT044). The library was validated with Qubit and real-time PCR for quantification and Bioanalyzer for size distribution detection. Quantified libraries were pooled and sequenced on Illumina platforms, according to the effective library concentration and amount of data.

Short-read sequences were mapped either to human GRCh38.104 or mouse GRCm39.104 reference sequence using the RNA-seq aligner STAR (v.2.5.0a)^68^. Differential gene expression analysis, statistical testing and annotation were performed in RStudio using DESeq2 (ref. ^69^). Gene Ontology and pathway analysis was performed using ShinyGO v.0.77 (refs. ^70^).

#### Western blot

HepG2 cells were washed in ice-cold PBS and lysed on ice in RIPA buffer (R0278, Sigma-Aldrich) supplemented with protease (1873580001, Roche/Merck) and phosphatase inhibitors (4906845001, Roche/Merck). Mouse tissue was collected at necropsy, snap frozen in liquid nitrogen or dry ice and stored at −80 °C before sample processing, when it was then homogenized in the RIPA-based lysis buffer described above using a FastPREP24 Classic tissue homogenizer. Extracts were cleared by centrifugation and the concentration of protein was determined using a Bio-Rad DC Protein Assay kit (5000116, Bio-Rad). Proteins were mixed to 1× with NuPAGE LDS Sample Buffer (4×) (NP0007, Thermo Fisher) and NuPAGE Sample Reducing Agent (10×) (NP0004, Thermo Fisher) and denatured for 5 min at 95 °C. Proteins were resolved by SDS–PAGE and transferred to nitrocellulose membranes using iBlot 2 Transfer Stacks (Invitrogen). Membranes were blocked in 5% (*w*/*v*) skimmed milk before applying antibodies. Membranes were developed using Immobilon Western Chemiluminescent Substrate (WBKLS0500, Millipore) and imaged by a ChemiDoc MP Imaging System (Bio-Rad).

#### Measurement of IL-1-β in liver lysates

Between 50 and 100 mg frozen liver tissue was lysed in 500 μl RIPA buffer (R0278, Sigma-Aldrich) supplemented with protease inhibitors (1873580001, Roche/Merck) using a FastPREP24 Classic tissue homogenizer in a metal bead lysing matrix. Lysate was cleared by centrifugation, diluted 1:4, and IL-1-β was quantified using a mouse IL-1-β Quantikine R&D ELISA kit, as per the manufacturer’s instructions.

#### Statistical analysis of mouse studies

Unless otherwise described above, studies were analysed using a Kruskal–Wallis test with Dunn’s multiple comparison test or ordinary one-way ANOVA with Holm–Šídak multiple comparison, based on the distribution of data and distribution of residuals. These were judged by visualization and formal statistical tests were not used. Body weight curve data were analysed by mixed-effects model with a post hoc Holm–Šídak test to account for repeated measures. No statistical methods were used to pre-determine sample sizes but our sample sizes are similar to those previously used^33^. Qualitative histopathological assessment was undertaken by an investigator blinded to genotype and other interventions, but aware of study design. No other data collection or analysis was undertaken blinded. Some values were excluded from statistical analysis and are detailed as follows. For CD68 immunostaining, a single outlier in the WT group was excluded (Fig. 6b) at ~6.7% total tissue. For liver triglyceride measurements in knock-in mice fed a low-fat diet (Extended Data Fig. 4i), two samples in the homozygous knock-in group failed basic QC as total glycerol measurements < free glycerol and were excluded. For serum cholesterol measurements in LXRα KO mice (Extended Data Fig. 6g), two WT mice had serum cholesterol levels of >13 mmol l^−1^ (13.13 and 13.15) with low HDL cholesterol levels (~0.3 mmol l^−1^) and were deemed outliers and excluded. For PSR staining in LXRα KO mice (Extended Data Fig. 6M), two heterozygous mice had high relative levels of fibrosis (>1.5%) and were deemed outliers and excluded.

Additional details of methods pertaining to human genetic follow-up and sensitivity analyses, replication of human genetic findings by an independently established computational pipeline, additional in vitro studies, histological preparation and analyses, flow cytometry and lipidomics are detailed in the Supplementary Methods.

### Reporting summary

Further information on research design is available in the Nature Portfolio Reporting Summary linked to this article.

## Supplementary information

Supplementary Information Supplementary Figs. 1–4 and Supplementary Methods.

Reporting Summary

Supplementary Tables 1–21 Supplementary tables and headers.

## Source data

Source Data Fig. 1 Normalized mutant activity data from luciferase assays statistical source data.

Source Data Fig. 2 Statistical source data.

Source Data Fig. 3 Statistical source data.

Source Data Fig. 4 Statistical source data.

Source Data Fig. 5 Statistical source data.

Source Data Fig. 6 Statistical source data.

Source Data Extended Data Fig. 2 Statistical source data.

Source Data Extended Data Fig. 3 Statistical source data.

Source Data Extended Data Fig. 4 Statistical source data.

Source Data Extended Data Fig. 5 Statistical source data.

Source Data Extended Data Fig. 6 Statistical source data.

Source Data Extended Data Fig. 7 Statistical source data.

Source Data Extended Data Fig. 7 Unprocessed western blots.

Source Data Extended Data Fig. 8 Statistical source data.

Source Data Extended Data Fig. 9 Statistical source data.

Source Data Extended Data Fig. 10 Statistical source data.

## Data Availability

The UKBB phenotype and whole-exome sequencing data described here are publicly available to registered researchers through the UKBB data access protocol. Information about registration for access to the data is available at https://www.ukbiobank.ac.uk/enable-your-research/apply-for-access. Data for this study were obtained under Resource Application no. 9905. Data from the Fenland cohort can be requested by bona fide researchers for specified scientific purposes via the study website (https://www.mrc-epid.cam.ac.uk/research/studies/fenland/information-for-researchers/). Data will either be shared through an institutional data-sharing agreement or arrangements will be made for analyses to be conducted remotely without the necessity for data transfer. Representative slide scans of liver sections are available for download at https://zenodo.org/records/12509122. The raw data for RNA-seq analyses are deposited to the Gene Expression Omnibus under accession IDs GSE273156 and GSE273158. Processed lipidomics data are available at 10.5281/zenodo.12790909 (ref. ^71^). The data used to generate the figures are available as source data or in associated supplementary tables. Source data are provided with this paper.

## Code availability

Rare variant association testing described in this paper was conducted using the MRC-EPID WES pipelines (https://github.com/mrcepid-rap/).
