# Supplementary material for: Damaging mutations in liver X receptor-α are hepatotoxic and implicate cholesterol sensing in liver health
Source: Nat Metab. 2024 Sep 25;6(10):1922–38. doi: 10.1038/s42255-024-01126-4 (PMC11496107; doi:10.1038/s42255-024-01126-4)
Supplement: Supplementary file 1 — Supplementary Figs. 1–4 and Supplementary Methods. [file 42255_2024_1126_MOESM1_ESM.pdf]

nature metabolism

Supplementary information https://doi.org/10.1038/s42255-024-01126-4

# Damaging mutations in liver X receptor-α are hepatotoxic and implicate cholesterol sensing in liver health

In the format provided by the authors and unedited

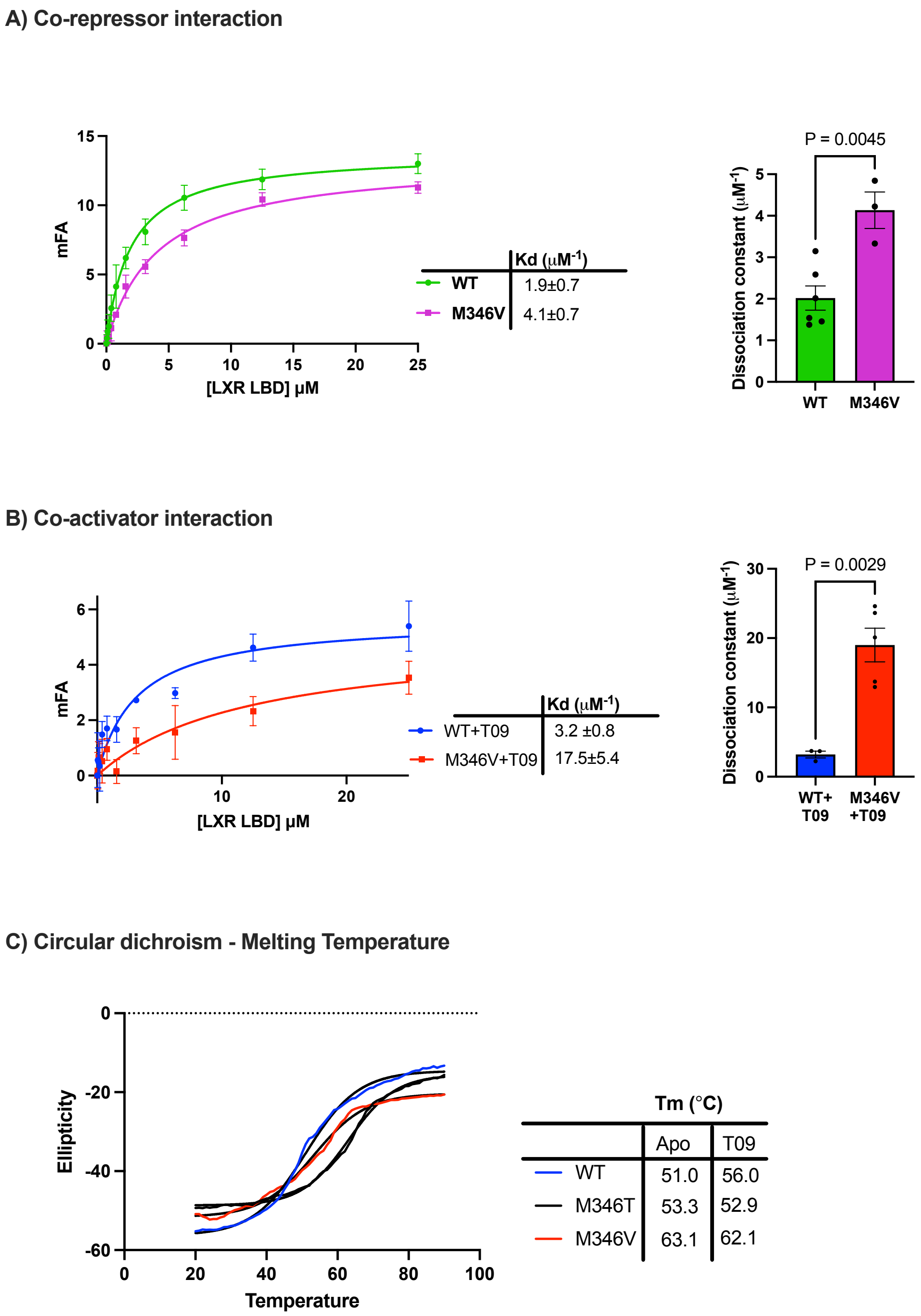


**Supplementary Figure 1. Biophysical study of gain of function mutants M346V/T LXRα by fluorescence anisotropy and circular dichroism.** Using co-repressor (A) and co-activator (B) peptides attached to a fluorophore, we studied the interaction of these peptides to the LXRα LBD mutant M346V and the thermal stability of M346V/T in vitro. A) Binding affinity in terms of dissociation constant of the WT (green) and M346V (magenta) LXRα LBDs to co-repressor peptide. B) Binding affinity in terms of dissociation constant of the WT (blue) and M346V (red) LXRα LBDs to co-activator peptide in the presence of agonist T09. C) Melting temperature of the WT (green) and M346V (magenta) M346T (purple) LXRα LBDs in the absence (apo) and presence of T09. In A and B, Kd ($uM^{-1}$) are reported in the tables +/- standard deviation. The bar charts represent mean±SEM, all p-values are two-sided P-values derived from an unpaired T-Test. A: N(WT)=6, N(M346V)=3, B:N(WT)=3, N(M346V)=5, where N refers to a fluorescence anisotropy experiment ran from an independent protein preparation.

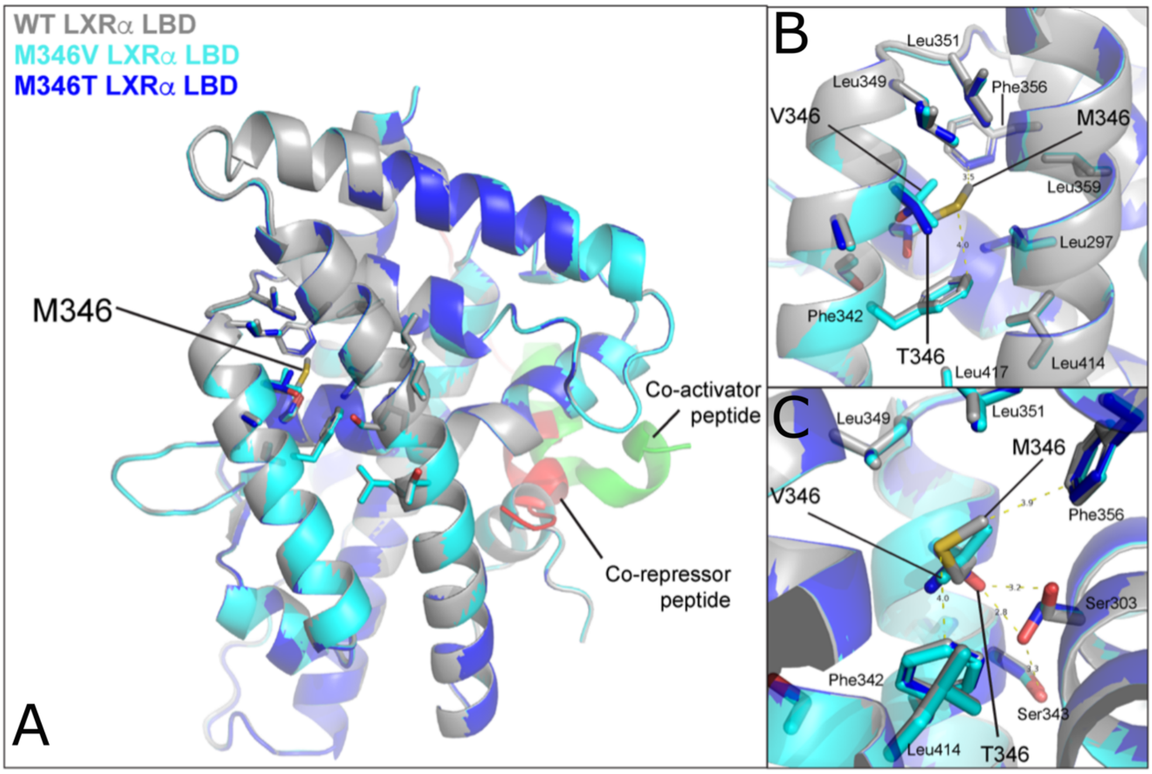


**Supplementary Figure 2. Structural models of the WT and M346V/T LXRα LBDs resulting from Alphafold. A)** Cartoon representation of the WT and M346V/T LXRα LBDs (grey, cyan and blue, respectively) models overlapping in order to highlight the differences in structure between the WT and the mutants as predicted by Alphafold. The amino acids in close proximity with the M346 (about 6 Å) are represented in sticks. The relative positions of co-repressor (red) and co-activator peptides (green) were modelled from structures PDB ID 1KKQ and 3PIQ, respectively. **B)** Close up view of the interactions between M/V/T346 and the amino acids surrounding represented as sticks. **C)** A different view of the M/V/T346 surrounding in order to highlight the potential hydrogen bonds formed by T346 with Serine 303 which would interact with Serine 343.

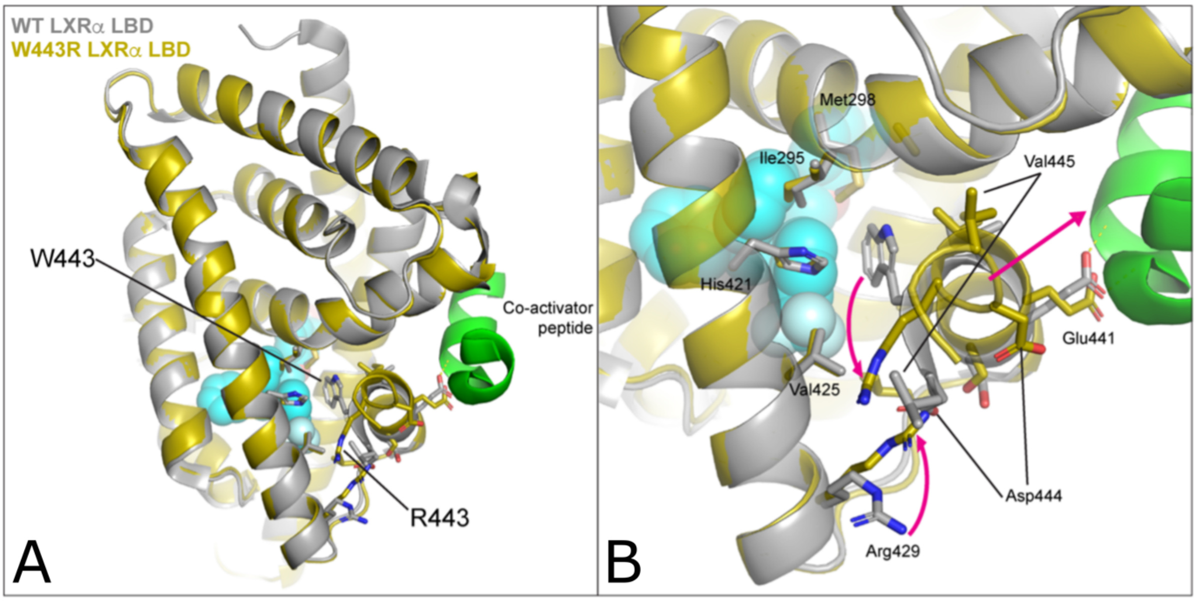


**Supplementary Figure 3. Differences between the WT and W443R LXRα LBDs Alphafold models.** Cartoon representation of the WT (grey) and W443R (yellow) LXRα LBDs models overlapping. The amino acids in close proximity with the W443 are represented in sticks. The relative positions of the agonist GW and co-activator peptide (green) were modelled from structures PDB ID 1KKQ and 3PIQ, respectively. The magenta arrows represent the relative movement of the W443 H12 position to the R443 H12 position according to the Alphafold model. Mutation W443R might potentially affect the interaction with the agonist and the active position of H12 which in turn would disturb the interaction with co-repressor and co-activator.

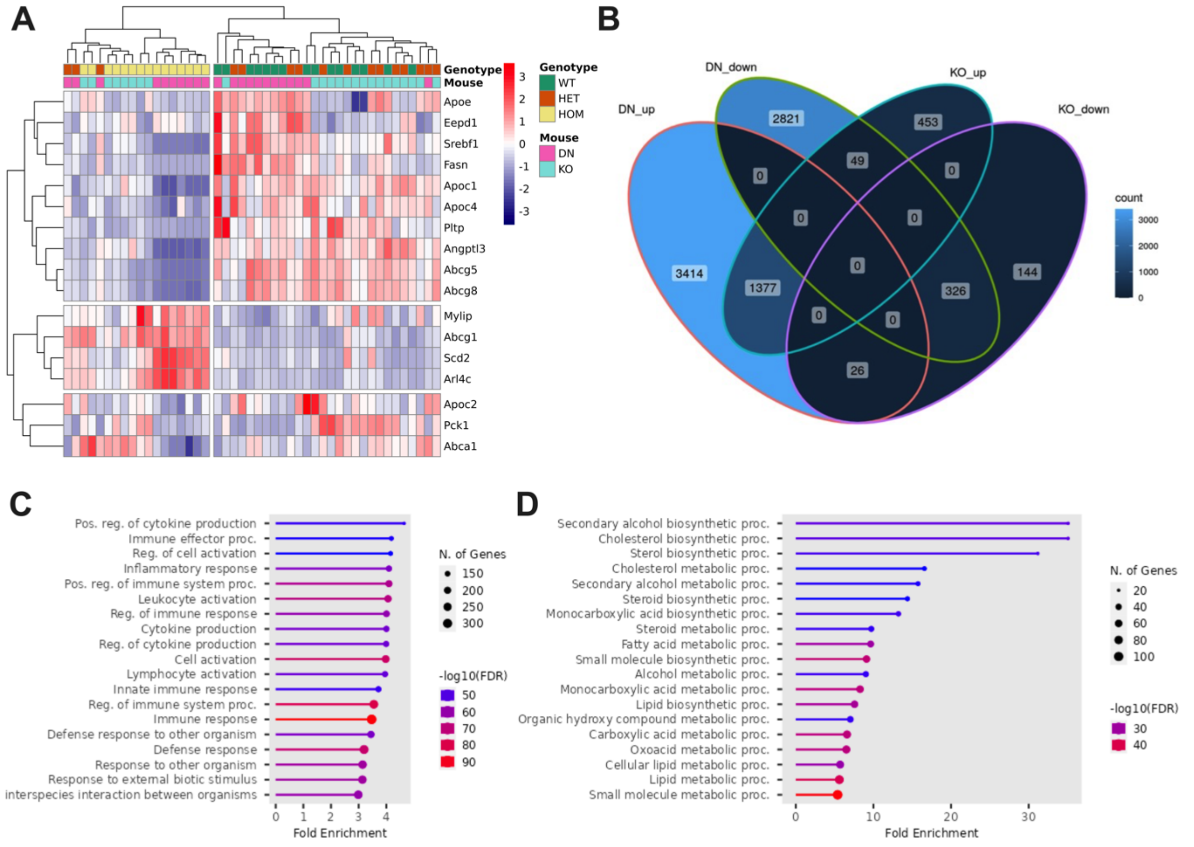


**Supplementary Figure 4. Liver RNA-seq analysis of male mouse livers at 16 weeks of age after 8 weeks on Western Diet.** 8 wildtype (LXRα$^{+/+}$), 8 heterozygous (LXRα$^{W441R/W441R}$) and 8 homozygous (LXRα$^{W441R/W441R}$) mice of the LXRα-W441R dominant-negative line (DN) were used. For the LXRα-KO (KO) mouse model, 8 wildtype (LXRα$^{+/+}$), 8 heterozygous (LXRα$^{+/-}$) and 8 homozygous (LXRα$^{-/-}$) mice were used. Heat map of RNA-seq expression data showing sample clustering based on mouse genes homologous to Reactome genes in '*NR1H2 and NR1H3-mediated signalling*' (R-HSA-9024446) (**A**). Venn diagram showing overlap of up and down-regulated genes in HOM vs WT in both DN and KO mouse lines (**B**). Gene Ontology (GO) pathway analysis of 1377 shared upregulated (**C**) and 326 downregulated genes (**D**) in both (LXRα$^{W441R/W441R}$ and LXRα$^{-/-}$) homozygous mouse models highlighting upregulation of inflammatory genes and downregulation of genes related to sterol and fatty acid metabolism.

**Supplementary Methods**

**Additional in vitro studies**

**Expression and purification of LXRα LBDs**

The human LXRα WT, M346V and M346T LBDs (residues 182-447) were cloned into a pGEX2T (GE Healthcare) vector containing an amino terminal GST purification tag followed by a TEV protease cleavage site. LXRα LBDs were expressed and purified as described previously (1). Briefly, E. coli Rosetta cells were grown at 37 °C in 2xTY media and induced with 40μM isopropyl-D-1-thiogalactopyranoside (IPTG). The pellet was lysed by sonication, bound to glutathione sepharose (GE Healthcare), and washed with a buffer containing 1xPBS, 0.5% Triton X-100, 1mM DTT. Then the GST tag was removed by incubation with TEV protease (100:1 molar ratio) overnight at 4°C. Eluted proteins were loaded onto 5-ml HiTrap Q HP Ion Exchange column (HiTrap Q HP IEX), previously equilibrated in low salt buffer (20mM Tris-HCl pH 7.4, 50 mM NaCl, 1mM DTT). The protein was eluted with a 50-500nM NaCl gradient at a flow of 1.5ml/min. Pooled fractions were buffer exchanged and concentrated in 25 mM Tris/HCl pH 7.5, 100 mM NaCl and 0.5 mM TCEP buffer for fluorescence anisotropy and circular dichroism assays.

**Fluorescence anisotropy**

Co-repressor and co-activator peptides were designed for use in the fluorescence anisotropy assay. An N-terminal FITC labelled 16-aa length peptide with sequence based on the interaction domain 1 of the SMRT corepressor protein (RID1 residues 2346-2360: Ac-STNMGLEAIIRKALMG-NH2), containing the corepressor NR recognition motif LxxxIxxx[I/L]. And a N-terminal FAM labelled 16-aa length peptide with sequence based on the second NR interaction box of GRIP1 coactivator protein (NID2 residues 686-700: Ac-KHKILHRLLQDSSC-NH2) containing the coactivator NR recognition motif LxxLL. Fluorescence anisotropy (FA) experiments were performed in black 384-well assay plates (Corning Life Sciences) as described previously [1]. Fixed concentration of SMRT and GRIP1 peptides (5nM) was used with increasing concentrations of LXRα LBDs (0-25μm) in a final volume of 20μl. For the assays in the presence of T09, increasing concentrations of the mixture protein:T09 in a 1:2 molar ratio were used. After incubation at 37 °C for 2 hours with slow shaking and centrifugation of the plates, the FA value was measured at each receptor concentration in a Victor X5 multilabel plate reader (Perkin Elmer, Singapore) using a 480-nm excitation filter and 535-nm emission filters to measure FITC and FAM emission. FA values were used to generate saturation binding curves that subsequently were used to calculate the equilibrium dissociation constant of the interaction (Kd), using the Prism software (Graphpad) and the nonlinear regression analysis.

**Circular dichroism**

Thermal unfolding of proteins was monitored by CD spectroscopy as described previously (1) using a Chirascan Spectrometer (Applied Photophysics) equipped with a temperature controller (Quantum Northwest TC125). CD spectra were measured from samples at 1mg/ml - apo - and molar ratio 1:2 (protein:agonist) when in the presence of T09 and GW.

**LXRα LBDs structure prediction**

Protein structure prediction was performed using AlphaFold2 [2], a machine-learning prediction of protein structure based on sequence and multiple sequence alignment.

**Generation of inducible HepG2 cell lines**

To generate the doxycycline-regulated lentiviral vector, human LXRα WT or W443R mutant genes were first cloned from pcDNA3.1-LXRα into the gateway-based entry vector pEN-Tmcs (ATCC-MBA-251, LGC Standard) by restriction cloning using SpeI and XhoI. Next, we cloned the LXRα

gene along with the doxycycline-inducible gene cassette into the lentiviral pSLIK-Neomycin vector by site-specific recombination using Gateway™ LR Clonase™ II Enzyme mix (11791020, ThermoFisher). All clones were confirmed by restriction digest screening and sequencing. Lentiviral particles were generated as previously described [3]. Briefly, 10 µg of pSLIK-LXRα-WT or pSLIK-LXRα-W443R plasmid, 7.5 µg of each of the packaging plasmids pMDLg/pRRE and pRSREV, and 5 µg of the pseudotyping pVSV-G plasmid were co-transfected into HEK293T cells in T75 flasks using CalPhos™ Mammalian Transfection Kit (631312, Takara Bio). The culture medium was replaced 12 hours after transfection with fresh media. Viral supernatants were harvested at 24, 48, and 72 hours after transfection and concentrated by centrifugation in Centricon Plus-70 Ultracel PL-100 (UFC710008, Millipore). Lentiviral particles were added at low MOI to HepG2 cells in the presence of 8 µg/ml of polybrene (H9268, Sigma-Aldrich) for 6 hours followed by selection in 2mg/ml G418 (10131035, Sigma-Aldrich) for 3 weeks. Stable cell lines were maintained in the presence of 0.5 mg/ml G418. To avoid early activation of transgene, both selection and maintenance media contained Tetracycline-free FBS (P30-3602, Pan Biotech). Doxycycline-inducible expression of LXRα was tested by immunofluorescence, qPCR and Western Blotting.

**Immunofluorescence microscopy**
Wild-type and transgenic HepG2 cell lines were treated with 1µg/ml doxycycline for 48 hours before two PBS wash and fixation in 4% formaldehyde/PBS for 15 minutes (28906, Thermo Fisher). Cells were permeabilised with 0.1% Triton/PBS for 10 minutes and blocked for 30 minutes in filter-sterilised 3% BSA/PBS. Slides were incubated with primary antibodies for 1 hour, followed by three PBS washes and 1 hour of secondary antibody incubation. After three further PBS washes, cells were mounted with ProLong™ Gold Antifade Mountant with DNA Stain DAPI (P36935, ThermoFisher).

**LDL-uptake assay**
HepG2 cell lines transduced with inducible $LXR\alpha^{WT}$ and $LXR\alpha^{W441R}$ expressing plasmids were seeded at 200,000 per well in 24 well plates and following day treated with 1µg/ml Doxycycline. After 24 hours, cells were serum-starved with 0.1% BSA, 1µg/ml Doxycyline with or without 1uM GW3965. After 24 hours, 2.5 ug/ml Dil-LDL (Generon, LDLD15-N-1) was added. After 4 hours of incubation, cells were lysed in RIPA buffer and lysate assessed for fluorescence (Excitation: 554nm, Emission: 571) using Spark 10M microplate reader (Tecan). Signal was normalised using total protein content. Analysis was conducted by three-way ANOVA with post-hoc testing undertaken using the holm-sidak method. *P<0.05, N=4 independent experiments.

## Additional human genetic analyses

### Sensitivity analyses
One loss of function variant, p.R415Q, is much more abundant than all other damaging variants studied (N=565) we therefore excluded this variant and repeated association testing using a variant mask including all other damaging variants, as described above. To exclude the possibility that our results were driven by known regional common variant signals, we ran genome wide association studies in UKBB participants of European ancestry for serum ALT, GGT, HDL and Triglycerides using linear-mixed models implemented in BOLT-LMM v2.3.2 [4]. Age, sex, genotyping chip and the first 10 genetic principal components were included as co-variates. We then fine-mapped the *NR1H3* (encoding LXRα) locus (11: 46790147 – 11:47790147) using

Genome-wide Complex Trait Analysis (GCTA) v1.92.0 [5] to identify conditionally independent signals. We used 'cojo-slct' to implement a stepwise model selection procedure, defining independent associated SNPs as those with an $R^2$<0.01 with a P-value of < 5 $x10^{-8}$. The refence sample used for linkage disequilibrium estimation in the fine-mapping analysis was a random sample of 25,000 unrelated European UKBB participants. SNP dosage was extracted for the lead SNP of each conditionally independent signal for each trait using Plink v.2.00 [6] and included in a joint linear model including carrier status of a rare damaging mutation in LXRα with age, age-squared, sex, first 10 principal components, exome sequencing batch and any phenotype specific co-variates as defined in **Supplementary Table 4**.

## Follow-up analyses

### Establishing dose response relationships between LXRα mutant activity and phenotypic effects

We ran marker-level association tests in UKBB for each phenotype of interest (as defined in supplementary table 3) using BOLT-LMM v2.3.6 adjusting for age, age-squared, sex, whole exome-sequencing batch and the first 10 principal components as described elsewhere [7]. We then queried resulting summary statistics for each tested variant and regressed the effect estimate for each variant against $Log_{10}$(mutant activity) in each assay with the inverse variance of the effect estimate used as weights in a linear model.

### Effect of rare damaging LXRα variants on circulating NMR-derived lipid metabolites in UKBB

Using, nuclear magnetic resonance (NMR) spectroscopy (Nightingale Health Plc.) -based metabolomics data available in UKBB, we extracted all non-derived lipid measures (all analytes in class Apolipoproteins, lipoprotein subclasses, lipoprotein particle sizes, fatty acids and other lipids, **Supplementary Table 10**) measured in 272,281 randomly selected participants at their baseline visit. We extracted metabolites and QC flags from the UKBB cloud-based Research Analysis Platform and removed known variations during technical handling using the R package *ukbnmr* (version 2 for the phase 2 release of UKBB data) [8]. The resultant values were inverse normal rank transformed and then association testing was undertaken using the 'Loss of function or dominant negative or protein truncating variant' mask as described above with age, age-squared, sex, wes batch and first 10 principal components. We considered an association significant if it past a Bonferroni corrected threshold of $5.7 \times 10^{-4}$.

### Effect of damaging rare damaging LXRα variants on liver disease and related traits in UKBB

To assess the effect of damaging mutations in LXRα on liver health we generated liver disease and liver fat outcomes as described in **Supplementary Table 5**. Alanine aminotransferase values were defined as normal/abnormal based on clinically recommended cut-offs [8]. Liver fat measurements were derived from available abdominal MRI imaging data - Fat referenced liver proton density fat fraction (FR-PDFF) and 10-point symmetric chemical-shift encoded acquisition liver proton density fat fraction (10P-PDFF) prioritising use of FR-PDFF measurements if both were available. Liver disease outcomes were defined using self-report information, hospital episode summary and death certificate data with qualifying diagnostic codes, procedural codes and self-reported conditions outlined in **Supplementary Table 5**. 'Any liver disease' and controls were defined as described in Verweij et al., [9] which excluded those with raised Alanine aminotransferase levels and those with ascites without clear evidence of a non-hepatic cause from the control group. Unlike Verweij et al., [9] we included self-report in our alcohol-related liver disease classification, as well as the ICD-10 code 'K70'. While the nomenclature and suggested diagnostic criteria for non-alcoholic fatty liver disease have recently been refined to describe a syndrome called metabolic dysfunction-associated steatotic liver disease the vast majority of cases available in UKBB will have been diagnosed before

dissemination of this information and therefore a non-alcoholic fatty liver disease (NAFLD) phenotype was derived based on ICD10 codes K75.8 and K76.0. Association testing was undertaken as described above.

**Effect of rare damaging LXRα variants on serum ALT according to genetically proxied liver fat**

**Derivation of liver fat polygenic risk score**

To derive the polygenic risk score 16 SNPs significantly associated with a composite phenotype of liver fat and NAFLD diagnosis in UKBB and the GOLD consortium at $P<5x10^{-8}$ in a published GWAS meta-analysis [10] were used. Genotypes for each of the SNPs were extracted for consenting individuals of european ancestry and genetic risk score derived with weightings using the effect size of each variant on liver fat measured in the Gold consortium [10]. The validity of this genetic proxy was assessed using a linear model with normalised (log-transformed) PDFF (derived as described above and in **Supplementary Table 5**) adjusted for PC1-10, age, age-squared, sex and method of assessment (10P-PDFF or FR-PDFF) and genotyping batch.

**Analysis stratified by genetically proxied liver fat**

To assess if the effect of damaging LXRα variants on alanine aminotransferase may be affected by propensity to liver fat accumulation we ran a linear model to assess for a statistical interaction between liver fat polygenic risk score and carriage of damaging LXRα variants on scaled, centred log2-transformed Alanine aminotransferase in consenting individuals of European ancestry in UKBB, adjusting for PC1-10, age, age-squared, sex and genotyping batch. We also plotted log2-normalised ALT according to liver fat PRS tertile and presence/absence of damaging LXRα variants.

**Replication via independently established computational pipelines**

**UKBB whole genome Sequencing processing and variant calling**

Whole-genome sequencing (WGS) data of the UKBB participants were generated by deCODE Genetics and the Wellcome Trust Sanger Institute as part of a public-private partnership involving AstraZeneca, Amgen, GlaxoSmithKline, Johnson & Johnson, Wellcome Trust Sanger, UK Research and Innovation, and the UKBB. The WGS sequencing methods and QC have been previously described [11, 12]. UK Biobank genomes were processed at AstraZeneca using the provided CRAM format files. A custom-built Amazon Web Services (AWS) cloud compute platform running Illumina DRAGEN Bio-IT Platform Germline Pipeline v3.7.8 was used to align the reads to the GRCh38 genome reference and to call small variants. Variants were annotated using SnpEff v4.3 [12] against Ensembl Build 38.92 [13]. We used *Peddy* and referenced 1000 genomes data [14] to classify EUR ancestry participants (peddy_prob>=0.90) removing those where peddy-derived principal components (PC) fell outside of 4 standard deviations from the mean over the first four PCs. Finally, we removed sex-discordant samples to leave 462,096 (94.2%) of unrelated EUR ancestry UKB participants for collapsing analysis.

**AstraZeneca Centre for Genomics Research (CGR) gene-level collapsing analysis pipeline**

According to the descriptions provided in previous studies [15], nine distinct models were established for collapsing non-synonymous variants, with one recessive and eight dominant models, along with an additional synonymous model as the negative control (**Supplementary Table 13**). These models were designed to consolidate functional variants that met specific

criteria, referred to as qualifying variants (QVs). QVs for each model were selected based on factors such as minor allele frequency (ranging from singleton to 0.1%), predicted consequences (e.g., protein-truncating variants, missense mutations), Rare Exome Variant Ensemble Learner (REVEL) and Missense Tolerance Ratio (MTR) scores [16, 17].

Liver function test quantitative biomarkers (ALT, AST, ALP, GGT) were transformed using rank-based inverse normalisation. Four binarized clinical outcomes related to liver disease and cirrhosis (any cause, alcoholic, non-alcoholic, cirrhosis [any cause]) were referred to the definitions in Verweij [9]. Both quantitative and binarized phenotypes were subsequently regressed against individual CGR collapsing analysis model carrier status, adjusting for age at recruitment, sex, and the first four genetic principal components (PCs), using linear regression and logistic regression models, respectively. The joint PDFF phenotype underwent two independent transformations: 1) log2-transformed with outlier removal (described above); 2) rank-based inverse normalisation. Both transformed phenotypes were then regressed against the individual CGR collapsing model, adjusting for age at the corresponding release instance, sex, PDFF method (10P-PDFF or FR-PDFF), and the first four genetic PCs.

**PTV-augmented collapsing model**

A composite model was created, combining carriers of experimentally identified LXRa functional variants (**Supplementary Table 21**) with individuals carrying other PTVs independently identified in the CGR collapsing *ptv* model. This joint model was subsequently regressed against all described binary and quantitative phenotypes above, adjusting for the relevant covariates.

## Additional methods related to mouse studies

### Sample processing and staining for flow cytometry

Livers perfused and then collected in ice cold PBS. Tissue was then cut into small pieces and mechanically dissociated using the FFX TissueGrinder using the liver protocol. The cell suspension was then passed through a 70 µm cell strainer and RBC lysed. Cells were then stained with LIVE/DEAD Fixable Scarlet (Invitrogen) and then Fc blocked (Biolegend). Cells were then stained with conjugated antibodies: EpCAM-PE (Biolegend, clone G8.8), CD45-BV510 (Biolegend, clone 30-F11), -PE-Cy7 (Biolegend, clone 390) and Ecadherin-BUV737 (Biosciences, clone DECMA-1) (all 1:100 dilution) at 4°C for 30 minutes. Cells were then analysed using a BD Symphony A5 flow cytometer using BD FACSDiveTM Diva software. Data were analysed using FlowJo software version 10.7.1.

### Histology and immunohistochemistry

Tissue samples were transferred from 10% formalin/PBS to 70% ethanol and processed into paraffin. 5-µm sections were deparaffinised, rehydrated, and then either stained with picrosirius red (PSR), haematoxylin and eosin (H&E) stain or were processed for immunohistochemistry (IHC). Briefly, endogenous peroxidase activity was blocked using a 0.6% hydrogen peroxide/methanol solution. Antigen retrieval was performed using 1mM EDTA for F4/80 and CD3 or antigen unmasking solution (H3301, Vector Laboratories) for αSMA, CD68 and 4-HNE. Primary incubations were performed with 1:100 F4/80 (D2S9R, Cell signalling technology), 1:200 CD3 (MCA1477, Bio-Rad), 1:1000 FITC conjugated αSMA (F3777 Sigma), 1:200 CD68 (OABB00472 Aviva Systems Biology) and 1:200 4-HNE (Abcam, ab46545), CK19 (Abcam, ab84632). Blocking was then performed using an Avidin/Biotin Blocking Kit (SP-2001 Vector Laboratories) followed by 20% swine serum in PBS. Sections were then incubated with primary antibodies diluted in 20%

swine serum overnight at 4°C, then a biotinylated goat anti-rabbit 1:400 (Vector Laboratories), biotinylated goat anti-fluorescein1:300 (BA-0601 Vector) or goat anti-rat 1:200 (STAR80B Serotec) and then incubated with Vectastain Elite ABC reagent (PK-7100 Vector Laboratories). Staining was visualised using DAB peroxidase substrate kit (SK-4100 Vector Laboratories), then counterstained with Mayers haematoxylin prior to mounting. Special stains (Reticulin, PAS/PASD, Victoria Blue) were undertaken by the Tissue Bank at Cambridge University Hospitals Foundation NHS Trust. For Reticulin staining, sections were oxidised in acidified potassium permanganate (0.15% sulphuric acid in 0.5% potassium permanganate) followed by sensitisation in 2.5% iron alum (ferric ammonium sulphate) before impregnation with an ammoniacal silver solution (prepared by reacting 10% silver nitrate with ammonia and 3.1% sodium hydroxide) for 8 seconds. Sections are then treated with 5% sodium thiosulphate for 2 minutes which prevents further silver reduction by light over time. PAS (Periodic Acid Schiff) staining was conducted using the Thermo Scientific Gemini AS using the PAS program. PASD staining was conducted via the same method after pre-treatment with 1% Amylase at 37 degrees Celsius. Victoria Blue staining: tissue sections were oxidised in acidified 0.5% potassium permanganate for 5 minutes, decolourised in 1% sodium metabisulphite before staining with Victoria Blue at 37 degrees Celsius for >4hours and then counterstained by nuclear fast red. Slides were scanned (Microscopy Zeiss Axioscan Z1 Slidescanner) and processed for collagen/fibrosis (Sirius staining in % area excluding vessels) and IHC (IHC staining in % area excluding vessels) quantification using HALO image analysis software (Indica Labs).

**Cholesterol crystal visualisation**

Frozen sections were prepared from flash frozen liver samples embedded in OCT from 3 randomly selected animals of each genotype for both the LXRα knockout and knockin western diet studies. The frozen sections were stained for haematoxylin and eosin (H&E), air dried and aqueously mounted in glycerol to allow detection of cholesterol crystals. Cross-polarised microscopy was used to assess for cholesterol burden using an ordinal grading system (0: absent 1: minimal 2: marked but patchy or moderate and diffuse 3: Marked and diffuse) by a histopathologist aware of study design but blinded to animal genotype.

**<u>Lipidomics analysis</u>**

**Liver lipid extraction and quantitation**

The protein-precipitation liquid extraction protocol described previously [18]. Briefly, 15-30 mg of liver tissue was homogenised in 650 µL of chloroform with single 5 mm stainless steel ball bearing in a VelociRuptor V2 Microtube Homogeniser (Scientific Laboratory Supplies). Then 100 µL of the lipid internal standard (1-10 µM in methanol), 100 µL of the carnitine internal standard (5 µM in methanol) and 150 µL of methanol was added to each sample, followed by thorough mixing. Then, 400 µL of acetone was added to each sample. The samples were vortexed and centrifuged for 10 minutes at ~20,000 g to pellet any insoluble material. The supernatant was pipetted into separate 2 mL screw cap amber-glass auto-sampler vials (Agilent Technologies). The organic extracts were dried using a Concentrator Plus system (Eppendorf) run for 60 minutes at 60°C.

The samples were reconstituted in 100 µL of 2:1:1 (propan-2-ol, acetonitrile and water, respectively), thoroughly vortexed and quantified by liquid chromatography with mass spectrometry detection (LC-MS) analysis, separately for cholesterol and cholesteryl-esters or full lipid profiles. The full chromatographic separation of cholesterol and cholesteryl-ester lipids was achieved using Waters Acquity H-Class HPLC System (Waters) with the injection of 5 µL onto a

Waters Acquity Premier UPLC® CSH C18 column; 1.7 µm, I.D. 2.1 mm X 50 mm, maintained at 55°C. Mobile phase A was 6:4, acetonitrile and water with 10 mM ammonium formate and 0.1% formic acid. Mobile phase B was 9:1, propan-2-ol and acetonitrile with 10 mM ammonium formate and 0.1% formic acid. For the analysis of the lipid profile, formic acid was excluded from the mobile phase as previously described [18]. The flow was maintained at 500 µL per minute through the following gradient: At startL 40% mobile phase B; at 1.5 minutes: 40% mobile phase B; at 8 minutes: 99% mobile phase B; at 10 minutes: 99% mobile phase B; at 10.10 minutes 40% mobile phase B; at 12 minutes, 40% mobile phase B. The sample injection needle was washed using 9:1, propan-2-ol and acetonitrile [strong wash] and 2:1:1 (propan-2-ol, acetonitrile and water) [weak wash]. The mass spectrometer used was the Thermo Scientific Q-Exactive Orbitrap with a heated electrospray ionisation source (Thermo Fisher Scientific). The mass spectrometer was calibrated immediately before sample analysis using positive and negative ionisation calibration solution. Additionally, the mass spectrometer scan rate was set at 4 Hz, giving a resolution of 35,000 (at 200 m/z) with a full-scan range of m/z 120 to 1,800 in positive mode.

The analytes detected at the correct retention time within a 5 ppm window of the theoretical m/z were integrated along with the appropriate internal standard. The area ratio between the internal standard and the analyte were then exported and were converted into absolute amounts normalised to weight (e.g. nmoles/mg) following quality checks and blank correction.

**Liver bile acids**
Bile acid concentrations were obtained from the lipidomics analysis data. Bile acid standards were analysed with the same method, showing deprotonated ions for cholic acid (m/z = 407.2803, Rt = 0.35), glycochenodeoxycholic acid (m/z = 448.3068, Rt = 0.64), glycocholic acid (m/z = 464.3018, Rt = 0.32), taurocholic acid (m/z = 514.2844, Rt = 0.27), deoxycholic acid ((m/z = 391.2854, Rt = 0.57) and lithocholic acid (m/z = 375.2905, Rt = 0.83). The method cannot distinguish taurocholic acid from muricholic acid (same m/z and Rt), and therefore data is presented as the combined signal of muricholic acid and taurocholic acid. Area under the curves for each signal were corrected against fatty acid internal standard (17:0d33) and corrected for the sample weight.

**Bioinformatic analyses of lipid profiles**
Lipidomics data was first identified and quantified using Thermo Scientific™ Xcalibur™ 4.1 Software and then exported for analysis using the *LipidR* package [19, 20]. If a lipid could not be detected in a sample, or quality checks were failed, values were set to half the minimum reported value. Data was then log transformed prior to analysis. Pairwise comparisons were undertaken between each genotype within each diet group to identify differentially regulated lipids using the 'de_design()' function in *LipidR* which uses a linear model to compare groups and is based on approaches developed for gene expression data [20, 21]. Lipid set enrichment analysis (LSEA) was then undertaken on the resulting dataset. LSEA is implemented in *LipidR* and is analogous to gene set enrichment though 'sets' in this implementation refer to lipid classes, chain length and saturation status. Enrichment scores are derived from a pre-ranked lipid list from differential expression and statistical significance of enrichment determined by permutation testing followed by correction for multiple testing [19, 20, 22, 23]. LSEA was undertaken across all contrasts tested to maintain control of Type 1 error rate. Heatmaps with hierarchical clustering of selected lipid classes were conducted using the *pheatmap* package in R.

**Supplementary References**


1. Romartinez-Alonso, B., et al., *Structure-Guided Approach to Relieving Transcriptional Repression in Resistance to Thyroid Hormone α.* Mol Cell Biol, 2022. **42**(2): p. e0036321.
2. Jumper, J., et al., *Highly accurate protein structure prediction with AlphaFold.* Nature, 2021. **596**(7873): p. 583-589.
3. Payne, F., et al., *Hypomorphism in human NSMCE2 linked to primordial dwarfism and insulin resistance.* J Clin Invest, 2014. **124**(9): p. 4028-38.
4. Loh, P.R., et al., *Efficient Bayesian mixed-model analysis increases association power in large cohorts.* Nat Genet, 2015. **47**(3): p. 284-90.
5. Yang, J., et al., *GCTA: a tool for genome-wide complex trait analysis.* Am J Hum Genet, 2011. **88**(1): p. 76-82.
6. Chang, C.C., et al., *Second-generation PLINK: rising to the challenge of larger and richer datasets.* Gigascience, 2015. **4**: p. 7.
7. Gardner, E.J., et al., *Damaging missense variants in <em>IGF1R</em> implicate a role for IGF-1 resistance in the aetiology of type 2 diabetes.* medRxiv, 2022: p. 2022.03.26.22272972.
8. Ritchie, S.C., et al., *Quality control and removal of technical variation of NMR metabolic biomarker data in ~120,000 UK Biobank participants.* Sci Data, 2023. **10**(1): p. 64.
9. Verweij, N., et al., *Germline Mutations in.* N Engl J Med, 2022. **387**(4): p. 332-344.
10. Chen, Y., et al., *Genome-wide association meta-analysis identifies 17 loci associated with nonalcoholic fatty liver disease.* Nat Genet, 2023. **55**(10): p. 1640-1650.
11. Shuwei, L., et al., *Whole-genome sequencing of half-a-million UK Biobank participants.* medRxiv, 2023: p. 2023.12.06.23299426.
12. Halldorsson, B.V., et al., *The sequences of 150,119 genomes in the UK Biobank.* Nature, 2022. **607**(7920): p. 732-740.
13. Zerbino, D.R., et al., *Ensembl 2018.* Nucleic Acids Res, 2018. **46**(D1): p. D754-D761.
14. Pedersen, B.S. and A.R. Quinlan, *Who's Who? Detecting and Resolving Sample Anomalies in Human DNA Sequencing Studies with Peddy.* Am J Hum Genet, 2017. **100**(3): p. 406-413.
15. Wang, Q., et al., *Rare variant contribution to human disease in 281,104 UK Biobank exomes.* Nature, 2021. **597**(7877): p. 527-532.
16. Ioannidis, N.M., et al., *REVEL: An Ensemble Method for Predicting the Pathogenicity of Rare Missense Variants.* Am J Hum Genet, 2016. **99**(4): p. 877-885.
17. Traynelis, J., et al., *Optimizing genomic medicine in epilepsy through a gene-customized approach to missense variant interpretation.* Genome Res, 2017. **27**(10): p. 1715-1729.
18. Jenkins, B., M. Ronis, and A. Koulman, *LC-MS Lipidomics: Exploiting a Simple High-Throughput Method for the Comprehensive Extraction of Lipids in a Ruminant Fat Dose-Response Study.* Metabolites, 2020. **10**(7).
19. Mohamed, A. and J. Molendijk, *Data Mining and Analysis of Lipidomics Datasets*. 2023.

20. Mohamed, A., J. Molendijk, and M.M. Hill, *lipidr: A Software Tool for Data Mining and Analysis of Lipidomics Datasets.* J Proteome Res, 2020. **19**(7): p. 2890-2897.
21. Smyth, G.K., *Linear models and empirical bayes methods for assessing differential expression in microarray experiments.* Stat Appl Genet Mol Biol, 2004. **3**: p. Article3.
22. Korotkevich, G., et al., *Fast gene set enrichment analysis.* bioRxiv, 2021: p. 060012.
23. Ramsey, S.A., et al., *Epigenome-guided analysis of the transcriptome of plaque macrophages during atherosclerosis regression reveals activation of the Wnt signaling pathway.* PLoS Genet, 2014. **10**(12): p. e1004828.
