# Supplementary material for: Damaging mutations in liver X receptor-α are hepatotoxic and implicate cholesterol sensing in liver health
Source: Nat Metab. 2024 Sep 25;6(10):1922–38. doi: 10.1038/s42255-024-01126-4 (PMC11496107; doi:10.1038/s42255-024-01126-4)
Supplement: Supplementary file 2 — Reporting Summary [file 42255_2024_1126_MOESM2_ESM.pdf]

nature portfolio

Corresponding author(s): Professor Stephen O'Rahilly
Dr Samuel M Lockhart
Dr Milan Muso

Last updated by author(s): 22/07/2024

Reporting Summary

Nature Portfolio wishes to improve the reproducibility of the work that we publish. This form provides structure for consistency and transparency in reporting. For further information on Nature Portfolio policies, see our Editorial Policies and the Editorial Policy Checklist.

Statistics

For all statistical analyses, confirm that the following items are present in the figure legend, table legend, main text, or Methods section.

n/a	Confirmed	
☐	☒	The exact sample size (*n*) for each experimental group/condition, given as a discrete number and unit of measurement
☐	☒	A statement on whether measurements were taken from distinct samples or whether the same sample was measured repeatedly
☐	☒	The statistical test(s) used AND whether they are one- or two-sided<br>*Only common tests should be described solely by name; describe more complex techniques in the Methods section.*
☐	☒	A description of all covariates tested
☐	☒	A description of any assumptions or corrections, such as tests of normality and adjustment for multiple comparisons
☐	☒	A full description of the statistical parameters including central tendency (e.g. means) or other basic estimates (e.g. regression coefficient) AND variation (e.g. standard deviation) or associated estimates of uncertainty (e.g. confidence intervals)
☐	☒	For null hypothesis testing, the test statistic (e.g. *F*, *t*, *r*) with confidence intervals, effect sizes, degrees of freedom and *P* value noted<br>*Give P values as exact values whenever suitable.*
☒	☐	For Bayesian analysis, information on the choice of priors and Markov chain Monte Carlo settings
☒	☐	For hierarchical and complex designs, identification of the appropriate level for tests and full reporting of outcomes
☐	☒	Estimates of effect sizes (e.g. Cohen's *d*, Pearson's *r*), indicating how they were calculated

Our web collection on statistics for biologists contains articles on many of the points above.

Software and code

Policy information about availability of computer code

Data collection	Western blot images were collected using BioRad ChemiDoc XRS+ and Image lab software (V6.1)
Data analysis	Graph Pad Prism version 9. RNASeq reads were aligned using STAAR (v2.5.0a) and analysed by DESeq2 in RStudio. Lipidomics data was first processed using Thermo Scientific™ Xcalibur™ 4.1 Software and then analysed in LipidR. PSR and Immunohistochemistry slides were quantified by HALO and HALO AI image analysis software (Indica Labs, both version 3.6.4134). Primary association testing was undertaken MRC-EPID WES Pipeline on the UKBB research access platform (https://github.com/mrcepid-rap/). For statistical analyses of mutant characterisation studies fixed effects models were generated using LmerTest (v3.1-3) and repeated measures ANOVAs Afex (v1.3-1). BD FACSDiveTM Diva v9.3.1 and FlowJo software version 10.7.1 were used for flow cytometry analysis

For manuscripts utilizing custom algorithms or software that are central to the research but not yet described in published literature, software must be made available to editors and reviewers. We strongly encourage code deposition in a community repository (e.g. GitHub). See the Nature Portfolio guidelines for submitting code & software for further information.

Data

Policy information about availability of data

All manuscripts must include a data availability statement. This statement should provide the following information, where applicable:

- Accession codes, unique identifiers, or web links for publicly available datasets
- A description of any restrictions on data availability
- For clinical datasets or third party data, please ensure that the statement adheres to our policy

Representative slidescans of liver sections are available for download at https://zenodo.org/records/12509122. The raw data for RNASeq analyses were deposited to the Gene Expression Omnibus under accession IDs - GSE273156 and GSE273158, processed lipodomics data is available at 10.5281/zenodo.12790909. The data used to generate our figures is available as source data, or in associated supplementary tables.
Access to the UK Biobank genotype and phenotype data are open to all approved health researchers, accessible through https://www.ukbiobank.ac.uk. Requests for anonymised human data may be addressed to the corresponding author - limitations on clinical data (which has to be anonymised) are designed to protect and respect patient and participant confidentiality.

Research involving human participants, their data, or biological material

Policy information about studies with human participants or human data. See also policy information about sex, gender (identity/presentation), and sexual orientation and race, ethnicity and racism.

Reporting on sex and gender	In human statistical genetics analyses conducted in UKBB genetic sex was included as a co-variate in all models, but sex-stratified analyses were not undertaken.
Reporting on race, ethnicity, or other socially relevant groupings	Our analysis was restricted to those of broadly European genetic ancestry defined using genetic principal component analysis.
Population characteristics	Age, age-squared, sex and the first 10 genetic principal components were used as co-variates in all our analyses. Where appropriate medication usage known to affect HbA1c or serum lipids was included as a co-variate.
Recruitment	Potential UK biobank participants were invited to participate by mail. ~500,000 people aged 40-69 between 2006 and 2010 across 22 centres distributed throughout the UK. Volunteer based recruitment is susceptible to 'healthy-volunteer' bias. This is a recognised limitation of the resource. We have used orthogonal strategies (e.g. mouse models) to substantiate our conclusions as well as to further our mechanistic understanding of the relationship between LXRa mutations and hepatotoxicity.
Ethics oversight	UK Biobank data have approval from the North West Multicentre Research Ethics Committee as a Research Tissue Bank. The Fenland study was approved by the Cambridge Local Research Ethics Committee (ref. 04/Q0108/19) and all participants provided written informed consent. All analyses reported here were conducted in accordance with relevant ethical guidelines.

Note that full information on the approval of the study protocol must also be provided in the manuscript.

Field-specific reporting

Please select the one below that is the best fit for your research. If you are not sure, read the appropriate sections before making your selection.

☒ Life sciences ☐ Behavioural & social sciences ☐ Ecological, evolutionary & environmental sciences

For a reference copy of the document with all sections, see nature.com/documents/nr-reporting-summary-flat.pdf

Life sciences study design

All studies must disclose on these points even when the disclosure is negative.

Sample size	Since we were interested in the clinical phenotypes associated with LXRa, we included all participants of european ancestry with proven damaging (or Protein Truncating, high-confidence LoF) variants and compared them to non-carriers. The novel mouse model generated in this study has not been phenotyped before and sample sizes for phenotyping studies were determined by availability of animals, researcher capacity and informed by previous studies on liver injury and cholesterol metabolism.
Data exclusions	CD68 immunostaining: A single outlier in the WT group was excluded (Figure 6b) ~6.7% total tissue. For liver triglyceride measurements in knock-in mice fed a low-fat diet (Extended Data Fig. 4i), two samples in the homozygous knock-in group failed basic QC as total glycerol measurements<free glycerol and were excluded. For serum cholesterol measurements in LXRα KO mice (Extended Data Fig. 6g), two WT mice had serum cholesterol levels of >13 mmol l−1 (13.13 and 13.15) with low HDL cholesterol levels (~0.3 mmol l−1) and were deemed outliers and excluded. For PSR staining in LXRα KO mice (Extended Data Fig. 6M), two heterozygous mice had high relative levels of fibrosis (>1.5%) and were deemed outliers and excluded.
Replication	The initial characterisation of LXRalpha-W441R mice on control and Western diet and LXRalpha-knockout mice were split into two cohorts and

Replication	aggregate results are presented, results are similar across cohorts consistent with successful replication. All functional assays were ran in duplicate and were repeated at least 3 times.
Randomization	For AAV8 rescue experiments mice were randomised to GFP or AAV8-NR1H3 injections. In all other animal studies the primary comparison was between genotypes, so allocation is not relevant. For the characterisation of the W441R knockin mice on control and western diet, cohorts of animals were arbitrarily assigned to one study or another, but this was not randomised.
Blinding	Qualitative histopathological assessment was undertaken by an investigator blinded to genotype, other interventions, but aware of study design. No other data collection or analysis was undertaken blinded. In the case of studies of W441R knock-in mice, blinding was not feasible due to the differences in weights of the homozygous knockin mice.

Reporting for specific materials, systems and methods

We require information from authors about some types of materials, experimental systems and methods used in many studies. Here, indicate whether each material, system or method listed is relevant to your study. If you are not sure if a list item applies to your research, read the appropriate section before selecting a response.

Materials & experimental systems

n/a	Involved in the study
☐	☒ Antibodies
☐	☒ Eukaryotic cell lines
☒	☐ Palaeontology and archaeology
☐	☒ Animals and other organisms
☒	☐ Clinical data
☒	☐ Dual use research of concern
☒	☐ Plants

Methods

n/a	Involved in the study
☒	☐ ChIP-seq
☐	☒ Flow cytometry
☒	☐ MRI-based neuroimaging

Antibodies

Antibodies used

Mouse Anti human LXR alpha antibody [PPZ0412] Abcam ab41902
Rabbit Anti human beta Tubulin antibody Abcam ab6046
Anti-GFP D5.1 Cell Signalling 2596S
Anti-rabbit IgG, HRP-linked Antibody Cell Signalling 70745
Anti-mouse IgG, HRP-linked Antibody Cell Signalling 70765
Goat anti-Rabbit IgG (H+L) Cross-Adsorbed Secondary Antibody, Alexa Fluor™ 488 Thermo Scientific A-11008
Goat anti-Mouse IgG (H+L) Cross-Adsorbed Secondary Antibody, Alexa Fluor™ 647 Thermo Scientific A-21235
PE anti-mouse CD326 (Ep-CAM) Antibody (clone G8.8) Biolegend 118205
Brilliant Violet 510™ anti-mouse CD45 Antibody (clone 30-F11) Biolegend 103137
PE/Cyanine7 anti-mouse CD31 Antibody (clone 390) Biolegend 102524
BD OptiBuild™ BUV737 Rat Anti-Mouse CD324 (E-Cadherin) (clone DECMA-1) BD Biosciences 752479
F4/80 (D2S9R) XP® Rabbit mAb Cell signalling technology 70076
Anti Human CD3 Antibody Bio-Rad MCA1477
Anti-Actin, a-Smooth Muscle (αSMA) - FITC monoclonal antibody Sigma-Aldrich F3777, clone 1A4
CD68 Polyclonal Antibody Aviva Systems Biology OABB00472
Anti-4 Hydroxynonenal (4-HNE) antibody Abcam ab46545
Goat anti-rabbit Vector Laboratories PI-1000
Biotinylated goat anti-fluorescein Vector Laboratories BA-0601
Goat anti-rat Bio-Rad STAR80

Validation

Validation data for the listed antibodies can be found on the manufacturers website as listed below:
Mouse Anti human LXR alpha antibody [PPZ0412] Abcam ab41902 - https://www.abcam.com/en-gb/products/primary-antibodies/lxr-alpha-antibody-ppz0412-ab41902
Rabbit Anti human beta Tubulin antibody Abcam ab6046 - https://www.abcam.com/en-gb/products/primary-antibodies/beta-tubulin-antibody-loading-control-ab6046
https://www.cellsignal.com/products/primary-antibodies/gfp-d5-1-rabbit-mab/2956Anti-rabbit IgG, HRP-linked Antibody Cell Signalling 70745
PE anti-mouse CD326 (Ep-CAM) Antibody (clone G8.8) Biolegend 118205 - https://www.biolegend.com/en-gb/products/pe-anti-mouse-cd326-ep-cam-antibody-4726
Brilliant Violet 510™ anti-mouse CD45 Antibody (clone 30-F11) Biolegend 103137 - https://www.biolegend.com/en-gb/products/brilliant-violet-510-anti-mouse-cd45-antibody-7995
PE/Cyanine7 anti-mouse CD31 Antibody (clone 390) Biolegend 102524 - https://www.biolegend.com/de-at/products/pe-cyanine7-anti-mouse-cd31-antibody-12996
F4/80 (D2S9R) XP® Rabbit mAb Cell signalling technology 70076 - https://www.cellsignal.com/products/primary-antibodies/f4-80-d2s9r-xp-rabbit-mab/70076
Anti Human CD3 Antibody Bio-Rad MCA1477 - https://www.bio-rad-antibodies.com/monoclonal/human-cd3-antibody-cd3-12-mca1477.html?f=purified#external-images
Anti-Actin, a-Smooth Muscle (αSMA) - FITC monoclonal antibody Sigma-Aldrich F3777, clone 1A4 - https://www.sigmaaldrich.com/GB/en/product/sigma/f3777
CD68 Polyclonal Antibody Aviva Systems Biology OABB00472 - https://www.avivasysbio.com/cd68-polyclonal-antibody-

oabb00472.html
Anti-4 Hydroxynonenal (4-HNE) antibody Abcam ab46545 - https://www.abcam.com/en-gb/products/primary-antibodies/4-hydroxynonenal-antibody-ab46545

Eukaryotic cell lines

Policy information about cell lines and Sex and Gender in Research

Cell line source(s)	HEK293, HEK293T and HepG2 cells were originally purchased from The European Collection of Authenticated Cell Cultures (ECACC).
Authentication	No authentication was performed, but both HEK293 and HepG2 cells presented with characteristic morphology known for this cells type.
Mycoplasma contamination	All cells used in this manuscript tested negative using EZ-PCR™ Mycoplasma Detection Kit (Biological Industries).
Commonly misidentified lines (See ICLAC register)	None.

Animals and other research organisms

Policy information about studies involving animals; ARRIVE guidelines recommended for reporting animal research, and Sex and Gender in Research

Laboratory animals	LXRa-KO mice and LXRa-W441R on C57Bl/6 background were used. The age and sex of animals in each study is clearly described in the figure legends. In the original characterisation of W441R knockin mice and LXR-KO mice, mice were ~8 weeks old at commencement of study. For the AAV-rescue experiments, animals were 10-12 weeks old. For the 45% HFD study mice were 16-17 weeks old.
Wild animals	No wild animals were used.
Reporting on sex	Sex for each study is clearly reported in each figure legend. Initial mouse studies with LXRalpha-KO and LXRalpha-W441R mice. The exogenous LXRalpha rescue experiment was performed and analysed together in both sexes to show the key findings of this study are relevant to both sexes.
Field-collected samples	No field-collected samples were used in this study.
Ethics oversight	All experiments with the LXRalpha-W441R mouse line were reviewed by the University of Cambridge Animal Welfare and Ethical Review Body (AWERB). All experiments with the LXRalpha-KO mouse were approved by the UCLA Institutional Animal Care and Research Advisory Committee. Experiments using flow cytometry to demonstrate the specificity of hepatocyte targeting of our AAV were approved by Newcastle University AWERB.

Note that full information on the approval of the study protocol must also be provided in the manuscript.

Plants

Seed stocks	*Report on the source of all seed stocks or other plant material used. If applicable, state the seed stock centre and catalogue number. If plant specimens were collected from the field, describe the collection location, date and sampling procedures.*
Novel plant genotypes	*Describe the methods by which all novel plant genotypes were produced. This includes those generated by transgenic approaches, gene editing, chemical/radiation-based mutagenesis and hybridization. For transgenic lines, describe the transformation method, the number of independent lines analyzed and the generation upon which experiments were performed. For gene-edited lines, describe the editor used, the endogenous sequence targeted for editing, the targeting guide RNA sequence (if applicable) and how the editor was applied.*
Authentication	*Describe any authentication procedures for each seed stock used or novel genotype generated. Describe any experiments used to assess the effect of a mutation and, where applicable, how potential secondary effects (e.g. second site T-DNA insertions, mosiacism, off-target gene editing) were examined.*

Flow Cytometry

Plots

Confirm that:

☒ The axis labels state the marker and fluorochrome used (e.g. CD4-FITC).

☒ The axis scales are clearly visible. Include numbers along axes only for bottom left plot of group (a 'group' is an analysis of identical markers).

☒ All plots are contour plots with outliers or pseudocolor plots.

☒ A numerical value for number of cells or percentage (with statistics) is provided.

Methodology

Sample preparation	Livers perfused and then collected in ice cold PBS. Tissue was then cut into small pieces and mechanically dissociated using the FFX TissueGrinder using the liver protocol. The cell suspension was then passed through a 70 µm cell strainer and RBC lysed.
Instrument	BD Symphony A5 flow cytometer
Software	Cells were then analysed using a BD Symphony A5 flow cytometer using BD FACSDiveTM Diva software. Data were analysed using FlowJo software version 10.7.1.
Cell population abundance	For each sample 1 million cells were counted using a haemocytometer and stained. Cell abundances are shown as a percentage of parent gate.
Gating strategy	Cell debris was excluded by FSC-H/SSC-A. Doublets were excluded by using FSH-H/FSC-A. Live/Dead exclusion was performed. EpCAM+ cholangiocytes, CD45+ immune cells, CD31+ endothelial cells, E-cadherin+ hepatocytes and Vitamin A+ fibroblasts were gated and GFP positivity assessed.

☒ Tick this box to confirm that a figure exemplifying the gating strategy is provided in the Supplementary Information.
