# Supplementary figures and images for: Damaging mutations in liver X receptor-α are hepatotoxic and implicate cholesterol sensing in liver health
Source: Nat Metab. 2024 Sep 25;6(10):1922–38. doi: 10.1038/s42255-024-01126-4 (PMC11496107; doi:10.1038/s42255-024-01126-4)

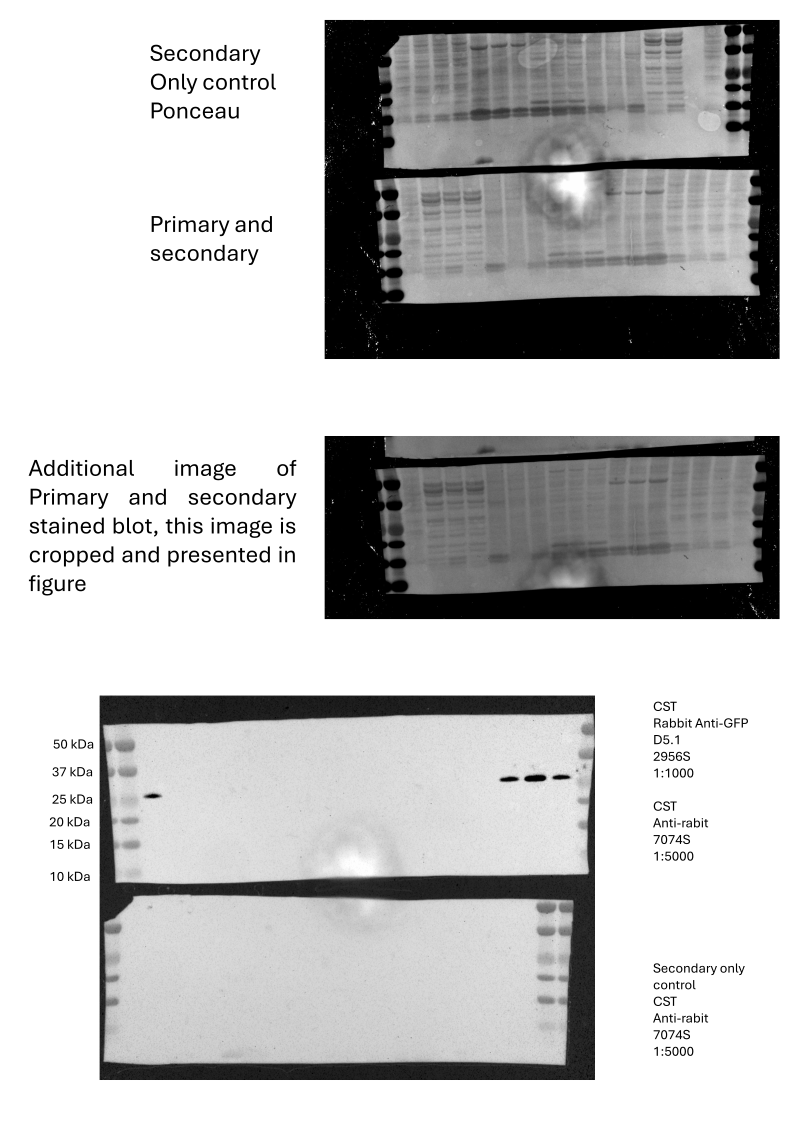

Supplement: Supplementary file 16 — Unprocessed western blots. [file 42255_2024_1126_MOESM16_ESM.tiff]
